# PROTACs: great opportunities for academia and industry

**DOI:** 10.1038/s41392-019-0101-6

**Published:** 2019-12-24

**Authors:** Xiuyun Sun, Hongying Gao, Yiqing Yang, Ming He, Yue Wu, Yugang Song, Yan Tong, Yu Rao

**Affiliations:** 1 Ministry of Education (MOE) Key Laboratory of Protein Sciences, School of Pharmaceutical Sciences, MOE Key Laboratory of Bioorganic Phosphorus Chemistry & Chemical Biology, Tsinghua University, Beijing, 100084 P. R. China; 2 Tsinghua-Peking Center for Life Sciences, Beijing, 100084 P. R. China; 3 School of Pharmaceutical Sciences, Zhengzhou University, Zhengzhou, 450001 China

**Keywords:** Chemical biology, Drug discovery

## Abstract

Although many kinds of therapies are applied in the clinic, drug-resistance is a major and unavoidable problem. Another disturbing statistic is the limited number of drug targets, which are presently only 20–25% of all protein targets that are currently being studied. Moreover, the focus of current explorations of targets are their enzymatic functions, which ignores the functions from their scaffold moiety. As a promising and appealing technology, PROteolysis TArgeting Chimeras (PROTACs) have attracted great attention both from academia and industry for finding available approaches to solve the above problems. PROTACs regulate protein function by degrading target proteins instead of inhibiting them, providing more sensitivity to drug-resistant targets and a greater chance to affect the nonenzymatic functions. PROTACs have been proven to show better selectivity compared to classic inhibitors. PROTACs can be described as a chemical knockdown approach with rapidity and reversibility, which presents new and different biology compared to other gene editing tools by avoiding misinterpretations that arise from potential genetic compensation and/or spontaneous mutations. PRTOACs have been widely explored throughout the world and have outperformed not only in cancer diseases, but also in immune disorders, viral infections and neurodegenerative diseases. Although PROTACs present a very promising and powerful approach for crossing the hurdles of present drug discovery and tool development in biology, more efforts are needed to gain to get deeper insight into the efficacy and safety of PROTACs in the clinic. More target binders and more E3 ligases applicable for developing PROTACs are waiting for exploration.

## Introduction

**Fig. 1:**
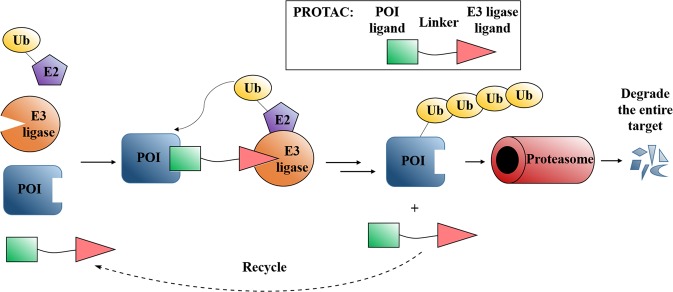
Mode of action of PROTACs.

PROteolysis TArgeting Chimeras (PROTACs) have become a promising and appealing technology for modulating a protein of interest (POI) by degradation.^1–41^ PROTACs are hetero bifunctional molecules that connect a POI ligand to an E3 ubiquitin ligase (E3) recruiting ligand with an optimal linker. Degradation is initiated when PROTACs promote the POI and E3 to form ternary complex.^28,42–49^ After that, subsequent POI ubiquitination happened when the ubiquitination machinery is brought in close proximity and then the ubiquitinated POI was recognized and degraded by the 26S proteasome, which is part of the ubiquitin-proteasome system (UPS) in eukaryotic cells (Fig. 1). PROTACs ally with the UPS system to achieve the regulation of protein levels. In other words, PROTACs represent a chemical knockdown strategy. In this review, we defined PROTACs as those compounds that meet the above standards. In addition PROTAC technology, there are some other types of protein degradation strategies including ‘molecular glue’,^50^ LYTAC,^51^ PhotoPROTAC,^52–55^ AUTAC,^56^ HomoPROTAC^57–61^ etc.^62^ Due to space limitations, this review will exclusively focus on PROTACs. PROTACs can degrade the entire protein, indicating that both the enzymatic activity and nonenzymatic functions would be deleted in the case of kinases. Meanwhile, the degradation induced by PROTACs are catalytic process due to their successful dissociation after promoting polyubiquitination of the POI, thereby providing great potential for allowing PROTAC action at very low doses. On the contrary, the inhibition process by traditional target is a competitive- and occupancy-driven event, while PROTAC induced degradation is iterative and therefore less susceptible to increases in target expression and mutations of the target protein. Therefore, with the above characteristics, PROTACs possess several advantages over traditional small molecules, including overcoming potential resistance to current therapeutic treatments.

Before the emergence of small-molecule based PROTACs, researchers employed different approaches to study intracellular protein function and target validation, such as the use of heat-shock protein 90 (HSP90) inhibitors and genetic fusion to the target protein. The destabilizing domain (DD), ligand-induced degradation (LID), and hydrophobic tagging (HyT) could all be fused to the target protein to induce target degradation. After that, peptidic PROTACs were developed as first-generation PROTACs, which provided the first proof of concept for PROTAC technology. Considering that the peptidic E3 recruiting moiety of the early PROTACs lacked good cell permeability, small-molecule based PROTAC were developed and achieved by Crews and coworkers in 2008. Inspired the first case of entirely small-molecule PROTAC targeting androgen receptor (AR) was realized by Crews’s group in 2008,^63^ a dramatic increase of targets were reported to be degraded by PROTACs.^2^ For the first small-molecule based E3 PROTACs induced degradation of AR successfully by recruiting the mouse double minute 2 homologue (MDM2) as E3 ligase and using a well-known MDM2-p53 PPI inhibitor, nutlin, as the E3 ligand.^64^ Although this first small-molecule PROTAC demonstrated good cell permeability, the potency was not satisfactory because micromolar concentrations were needed to achieve the degradation of AR. During the same time, the cellular inhibitor of apoptosis protein 1 (cIAP1) was used as an E3 in the design of PROTACs because bestatin methyl esters exhibited good binding affinity to cIAP1 and promoted its autoubiquitination and degradation.^65^ The first PROTAC recruiting cIAP1 was developed by Hashimoto and coworkers for degrading targeting the cellular retinol- and retinoic acid-binding proteins (CRABP-I and II).^65^ Degraders recruiting IAP were named specific and nongenetic IAP-dependent protein erasers (SNIPERs).^66–76^ Later, von Hippel-Lindau ligands used for PROTAC design were identified by the Ciulli laboratory.^77–79^ Concurrently, it was found that the E3 cereblon (CRBN) was the molecular target of the immunomodulatory drugs (IMiDs), such as thalidomide, pomalidomide, and lenalidomide.^80–82^ Before that, IMiDs were reported as a promoter of recruiting neosubstrates, such as Ikaros, Aiolos, and casein kinase 1A1 (CK1a) for ubiquitination and subsequent proteasomal degradation. Based on the above advances, scientists have disclosed a large number of PROTACs to degrade different POIs for the treatment of different diseases, including cancers, viral infections, immune disorders, and neurodegenerative diseases. The scope that can be touched by PROTACs is still expanding with dramatic speed and impressive achievements.

**Fig. 2:**
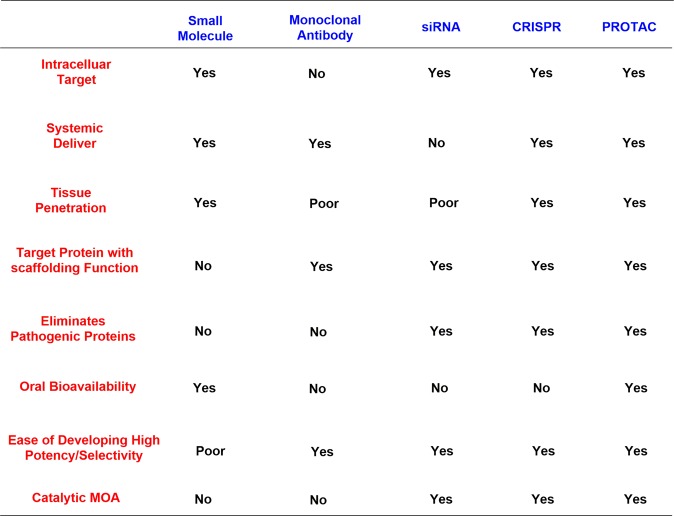
Comparisons of PROTACs with other therapeutic modalities.

As new and promising techniques, PROTACs show great opportunities for the following aspects (Fig. 2). First, PROTACs have demonstrated a particular sensitivity to drug-resistant targets. Chemotherapy has traditionally been the major therapy for cancer treatments traditionally. Clinical applications have been hindered by the acquired resistance to chemotherapy drugs and have resulted in relapse of the disease.^83–86^ With research progress on new targets and novel drug discovery technologies, another powerful strategy appeared to inhibit the functions of oncogenic proteins or receptors by small molecules directly and specifically. Notably, the dramatic developments of kinase inhibitors have been achieved in the past few decades that have providing amazing therapeutic effects in clinical practice and have greatly prolonged the patient survival.^87^ Regrettably, fast growing of resistance to these kinase inhibitors was onset after the first euphoric period, resulting in consequent relapse, especially for some patients with advanced cancer.^88–90^ Drug-resistance was an incredibly daunting problem for current studies, particularly for cancer research. Alterations in the drug target, activation of pro-survival pathways and ineffective induction of cell death, etc. contributed to the acquirements of drug resistance.^91–93^ The present approach for addressing the emergence of drug resistance is to develop new inhibitors targeting mutant kinases generation after generation, leading to a tremendous expense of resources and time.^94,95^ Surrounded by the growing resistance caused by target treatments, the immune system becomes another treasured strategy for discovery of more effective tactics for cancer treatment.^96^ A number of tumors have been conquered by the antibodies through blocking the immune checkpoints PD-1 (programmed death 1) and CTLA-4 (cytotoxic T lymphocyte–associated protein 4). Several response biomarkers to immune-checkpoint inhibitors have been identified, including checkpoint-ligand expression, DNA-repair deficiency, and mutational burden. These biomarkers are beneficial for predicting the response of the patients treated by checkpoint inhibition. However, it was still uncertain to predict the degree, duration of response or their absence does not preclude a response. Due to the complex interactions between the immune system and advanced malignancies, challenges are remained to develop biomarkers for immunotherapy. Moreover, the resistance to immunotherapy has been found in clinical observations and relative studies, including low intrinsic, adaptive resistance, and high intrinsic, adaptive resistance.^97^ Thus, it is extremely urgent and important to develop novel technologies to overcome the ever-growing drug resistance of cancer. As mentioned above, PROTACs have unique characteristics which provide surprising effects. Since PROTACs influence protein function by eliminating the entire target to delete the whole functions of the targets, including enzymatic activity and nonenzymatic functions, PROTACs can address the potential resistance faced by current therapeutic treatments. In addition, PROTACs are less susceptible to increases in target expression and mutations in the target protein because only low doses of PROTACs are needed because they act catalytically. Presently, several drug-resistant targets have been solved with impressive inhibition activity, such as Bruton’s tyrosine kinase, the androgen receptor and the estrogen receptor. Second, PROTACs have the potential to target undruggable targets. As is well known, there are only 20–25% of all protein targets are currently in research for drug discovery, including enzymes, GPCRs, nuclear hormone receptors, and ion channels. The remaining protein targets are still unexplored. The focus on making more potential drug targets accessible is increasing, which has been sped up by PROTACs due to their unique mode of action motion. Signal transducer and activator of transcription 3 (STAT3) is a key factor for cell survival, proliferation, angiogenesis, metastasis, and chemotherapy resistance. Thus, blocking the STAT3 activity is a rational idea to develop as new therapeutic strategies, which has been pursued for many years. Little success has been obtained by blocking STAT3 directly since the difficulty of hunting an obviously druggable site from the STAT3.^98^ Although a number of small-molecule tool compounds for treating cancer in preclinical assays have been reported, all were hard to push their applications further due to micromolarpotencies and lack of specificity for STAT3 over other STAT proteins. Another approach to inhibiting STAT3 activity is decoy oligodeoxynucleotides (dODNs) based on the consensus promoter sequence that STAT3 recognizes. dODNs bind to activated STAT3 dimers and sequester them in the cytoplasm. However, the naked dODNs applications in vivo have been limited due to the poor penetrability, and their rapid degradation by serum nucleases. In contrast, PROTACs have disclosed obvious advantages in blocking the activity by degrading STAT3. STAT3 degraders have been developed by the Wang group and have demonstrated high efficacy both in vitro and in vivo. The expression of KRAS alone cannot drive tumorigenesis because it cannot promote the activation of K-Ras. K-Ras can be activated by several approaches, such as binding to guanosine triphosphate, or becoming activated by the activation of cell surface receptors, including receptor tyrosine kinases (RTKs). Except for activation by proteins, K-Ras also keeps an constitutive activation mode when mutations occurred in critical codons, characterized with G12A, G12C, G12D, G12S, G12V, G13C, and G13D in high frequency, and other low-frequency mutations.^99^ These mutations induced cancers by interfering with guanosine triphosphate hydrolysis to activate K-Ras constitutively. The constitutively active state of K-Ras causes great difficulty for drug discovery. In contrast to traditional inhibitors, PROTACs may block the activity of K-Ras by degrading proteins. Third, PROTACs can influence the nonenzymatic function by degrading the whole protein. Traditional small-molecule drugs generally exert their functions by eliminating the enzymatic activity of their targets. Focal adhesion kinase (FAK) plays a critical role in tumor invasion and metastasis with the cooperation of both kinase activity and scaffold function for several signaling proteins simultaneously. However, inhibition of FAK though modulation of FAK kinase activity has not been successful in clinical studies. Therefore, PROTACs offer the possibility to simultaneously block the kinase signaling and scaffolding capabilities of FAK. Crews and coworkers reported a selective and potent FAK degrader, which showed obvious advantages over defactinib, a FAK inhibitor in clinic, both in FAK activation and FAK-mediated cell migration and invasion.^100^ These data indicated the potential that PROTACs could expand the druggable space and control protein enzymatic and non-enzymatic functions that are not easily addressed by traditional small-molecule inhibitors. Many other advantages are presented by PROTACs. For instance, PROTACs afford better selectivity compared with traditional inhibitors. Ibrutinib can bind a series of BTK homologs, including BTK, ITK, and TEC, while PROTACs derived from ibrutinib only degrade BTK. In addition, PROTACs can be a novel technology for wide applications in other research areas. Researchers have broadened PROTACs for the treatment of immune disorders by targeting IRAK4, sirtuin, and PCAF/GCN5. Viral infections and neurodegenerative diseases can also be affected by PROTAC by targeting NS3/4A and Tau respectively. Last but not least, PROTACs may present new and interesting biology as a chemical knock-down approach in a fast and reversible way. It was a powerful strategy for investigating the functional consequence of the loss of a target gene by generating animal models with protein deletions. Traditionally, animal models are constructed by genetic modification mechanisms, such as RNA interference, transcription activator-like effector nucleases (TALEN) and clustered regularly interspaced short palindromic repeats (CRISPR)-Cas9 genome editing. However, these approaches have failed to achieve acute and reversible changes, which makes the research more challenging due to the long duration and high cost of genetic modifications, particularly in nonhuman primates. It was usually misleading for gene knockout models considering the complication of potential genetic compensation and/or spontaneous mutations. Moreover, the chance for animals to activate compensatory mechanisms has been reported to increase in the long term and may mask the phenotypes. In addition, for those genes that are lethal when they are deleted during embryonic development, appropriate tools are still urgently demanded. PROTACs are a chemical knockdown approach of targeted proteins in a novel, fast and effective way to generate protein depletion models. Given the success of PROTACs on different targets, a few factors including the POI binder, linker and E3 ligase, are critical for developing effective degraders.^17,101–103^ The selection of the proper POI ligands and E3 ligases is the first and most crucial step. Then, the binding position used for linking the E3 ligase ligands is important in the design, which can be predicted by the binding mode of the POI and its ligand. In addition, the length and flexibility of the linker between the two ligands binding to the POI and E3 ligase can significantly influence the potency and selectivity. However, it is still difficult to achieve rational design at the present stage. More research efforts towards a deeper and more comprehensive understanding of PROTACs will be needed for rational design in the near future.

Although PROTACs depict a promising technology for a variety of aspects, including drug discovery and answers to biological issues, challenges exist for PROTACs in the future. It is essential to find optimal ligands to successful design PROTACs, particularly for protein–protein interactions. How to obtain deep insight on degradation activity and selectivity and how to design compounds based on this understanding are another question for PROTACs. The in vivo efficacy and pharmacokinetic and pharmacodynamic characteristics are not very clear in clinical practice. In addition, more than 600 E3 ubiquitin ligases were encoded by the human genome. However, only a handful of E3 ligases (VHL, CRBN, IAPs, and MDM2) can be recruited to degrade target proteins within cells in present chimeric small molecules that until now. specific ligands for many other E3 ligases are still lack thereby complicates the extended application of this protein knockdown technology. Another potential challenge for PROTAC development is resistance in degrader-treated cells.^104^ It has been reported that PROTAC could cause resistance by genomic alterations in the core components of E3 ligase complexes. In addition, thalidomide derivatives could also induce the degradation of IKZF1, IKZF3 and GSTP1, indicating that PROTACs with high selectivity are needed to avoid the possible degradation of the proteins resulting from the CRBN ligands themselves.^105^

**Fig. 3:**
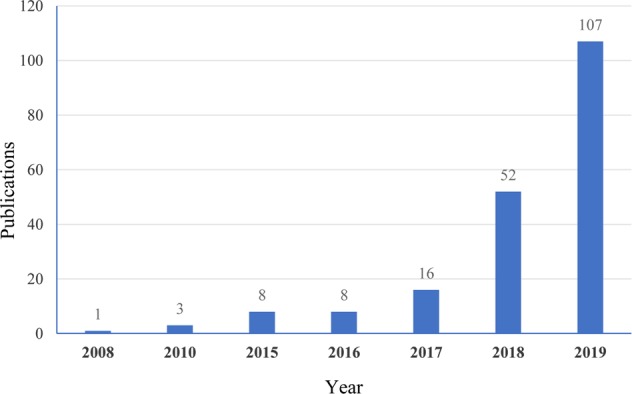
Publications on PROTACs in recent years.

**Fig. 4:**
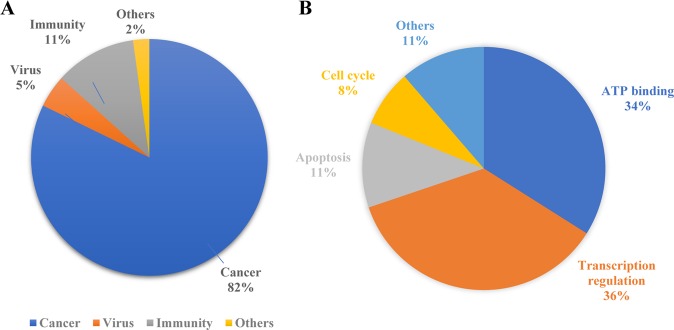
Distribution of PROTACs. **a** Efficacy of PROTACs in different diseases. **b** Efficacy of PROTACs in different biological processes.

This exciting topic has already been covered in some reviews and accounts. As the PROTAC field quickly grows and many new studies have been documented in recent literature (Fig. 3), we will give a comprehensive update to cover recent research advances in the area. Herein, we summarized as many of the targets of PROTACs as we could. More than 40 targets have been degraded by PROTACs to date. Following the criteria, such as disease field and drug-target class (Fig. 4), we will introduce the degraders in alphabetical order of the targets one by one. Considering the amazing attraction and remarkable process of PROTACs, we hope this review can be a complimentary summary to the other reviews in the field of protein degradation.

PROTAC can bind POI and recruit E3 ligase for ubiquitination and subsequent degradation of the entire protein. After that, PROTAC could dissociate from the ternary complex for the next degradation cycle. POI: protein of interest; Ub: ubiquitin.

## PROTACs targeting cancer-related targets

### AHR

The aryl hydrocarbon receptor (AHR) is a ligand-activated transcription factor that belongs to the bHLH/PAS (basic helix-loop-helix/Per-Arnt-Sim) family of chemosensors.^106,107^ It mediates many of the toxicities and carcinogenic effects from environmental carcinogens including chloracne, wasting, teratogenicity, immunotoxicity, tumor promotion, and carcinogenicity. AHR binds its ligand like a polycyclic aromatic hydrocarbon and initiates xenobiotic-metabolizing enzymes such as cytochrome P450, followed by the generation of DNA adducts. Although AHR-associated physiological disorders have been received much attention, the role of AHR in those pathological processes has yet to be clearly studied due to the lack of powerful chemical probes.^108^

**Fig. 5:**
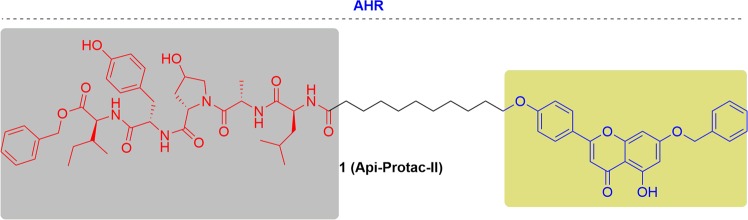
Representative PROTAC of AHR.

Kim’s group recently revealed that apigenin, a natural product found in fruits, plants and honey, interacted directly with AHR (Fig. 5). They then envisioned that apigenin-based PROTACs might be useful molecular probes for studying AHR biology.^108^ To design AHR-targeting PROTACs, they first selected a position on apigenin to attach the VHL ligand. After a modification of the free hydroxyl groups by acetyl groups, it was found that all apigenins maintained the ability to inhibit AHR after acetylation. They linked a pentapeptide fragment derived from hypoxia inducing factor (HIF)-1α, a ligand of VHL, to apigenin or benzylated apigenin. Api-Protac-II effectively induced the degradation of AHR, while degradation was not observed with Api-Protac-I. They also examined the degradation of enhanced green fluorescent protein (eGFP)-fused AHR. Their results suggested that Api-Protac-II could be used as a probe for studying AHR biology.

### ALK

Anaplastic lymphoma kinase (ALK) is a tyrosine kinase of the insulin receptor (IR) kinase subfamily.^109^ Oncogenic activation of ALK is highly related to the occurrence and development of many human cancers, including diffuse large B cell lymphoma (DLBCL), anaplastic large-cell non-Hodgkin's lymphoma (ALCL), esophageal squamous cell carcinoma (ESCC), inflammatory myofibroblastic tumor (IMT), non-small cell lung cancer (NSCLC), renal cell cancer (RCC), neuroblastoma, thyroid cancer, ovarian cancer, colon carcinoma, and breast cancer.^110–115^ ALK is activated mainly through three different mechanisms and chromosomal translocations are the most common genetic alterations. Nearly 30 various types of an ALK fusion protein have been identified, and among them, the fused forms of NPM-ALK, EML4-ALK, KIF5B-ALK, and TGF-ALK have been commonly found in different types of cancer, including ALCL, NSCLC, and DLBCL.^116^ Substitution mutations are the second mechanism of ALK activation, and point mutations in the kinase domain (F1174L and R1275Q) are most frequently observed in neuroblastoma.^117^ Gene amplification and overexpression is the third mechanism of ALK activation, which has also been reported in many types of human cancers.^118,119^ To date, five ALK inhibitors, including alectinib, brigatinib, ceritinib, crizotinib, and lorlatinib have been approved by the FDA for the therapy of ALK-positive NSCLC.^120^ Despite an initial response to these inhibitors, drug resistance has been observed within 1–2 years in the majority of patients.^120,121^ Thus, new therapeutic approaches are urgently needed to overcome drug resistance.

**Fig. 6:**
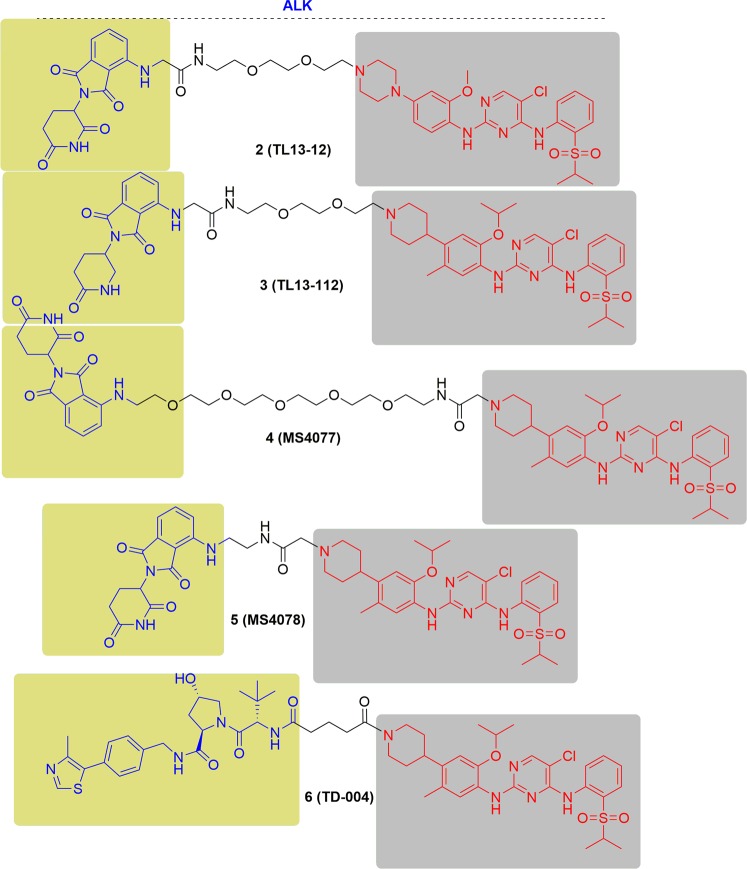
Representative PROTACs of ALK.

In the year of 2018, two ALK PROTACs (TL13–12 and TL13–112) were developed by Nathanael S. Gray group. These two PROTACs were conjugated with ALK inhibitors (ceritinib and TAE684) and pomalidomide by different lengths of polyethylene glycol (PEG) likers (Fig. 6).^122^ These PROTACs induced the potent knockdown of ALK in NSCLC cells H3122 (EML4-ALK), ALCL cells Karpas 299 (NPM-ALK), ALCL cells SU-DHL-1 (NPM-ALK) and NB cells (F1174L/R1275Q ALK), which sustained the ALK downstream signaling inhibition at the same time. In addition, these PROTACs promoted other kinase degradation (FAK, Aurora A, FER, and RSK1) by proteomic profiling analysis.

In the same year, Jian Jin and coworkers reported two ALK-targeting PROTACs, MS4077 and MS4078, through connecting ceritinib and pomalidomide with two distinct linkers (Fig. 6).^123^ In addition to degrading NPM-ALK and EML4-ALK, MS4077 and MS4078 could inhibit ALK and STAT3 phosphorylation in concentration- and time-dependent manners in both SU-DHL-1 (ALCL cells harboring NPM-ALK) and NCI-H2228 cells (NSCLC cells harboring EML4-ALK). In addition, MS4077 and MS4078 showed potent antiproliferative activity in SU-DHL-1 cells. Furthermore, MS4078 displayed good plasma exposure in a mouse pharmacokinetic study, and the tested mice were well tolerated a dose of 50 mpk (mg/kg).

Subsequently, Jong Yeon Hwang and his coworkers reported TD-004, which consisted of ceritinib and a VHL E3 ligase ligand (Fig. 6).^40^ TD-004 exhibited excellent ALK degradation and cell growth inhibition of SU-DHL-1 and H3122 cells. Furthermore, TD-004 dramatically decreased tumor size in a H3122 xenograft mouse model in vivo.

### AR

Disorder of the androgen receptor is the main driving force for prostate cancer.^124^ The first-line drugs treating prostate cancer were competitive antagonists, such as enzalutamide, which could inhibit the transcriptional activity of AR. However, after long term exposure of these antagonists, drug resistance would be developed eventually for the majority of patients.^125^

**Fig. 7:**
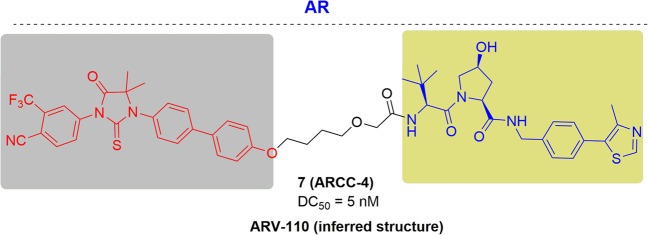
Representative PROTACs degrading drug-resistant AR.

Derived from enzalutamide, a PROTAC targeting AR named ARCC-4 was reported by Crews and his colleagues in 2018^126^ (Fig. 7). ARCC-4 is a highly efficient degrader with low-nanomolar degrading activity and its DC_50_ (half-maximal degradation concentrations) was 5 nM. Moreover, ARCC-4 showed inhibitory proliferation effects on prostate tumor cells, and ability of degrading the clinically relevant mutant androgen receptor. In addition, different cellular models of prostate cancer drug resistance were used to a parallel compare enzalutamide and ARCC-4. For instance, ARCC could decrease the AR level (~3.5-fold at 10 μM) in LNCaP cells engineered to overexpress the mutant AR-F876L (LNCaP/F876L), while AR in the cells treated by the enzalutamide increased substantially (~17.5-fold at 10 μM). Other AR point mutations in patients exposed to AR-targeted therapies were also degraded efficiently, including H874Y, M896V, T877A, L702H. Therefore, ARCC-4 offered a better antiproliferative effects in an AR mutant environment while enzalutamide failed.

Arvinas developed another PROTAC targeting the AR (ARV-110), which exhibited high potency of degrading both AR and AR mutants after oral administration (Fig. 7). ARV-110 could degrade 95–98% of the AR in a variety of cell lines commonly used in prostate cancer studies. ARV-110 demonstrated comparable efficacy at lower doses compared to enzalutamide in the wild-type AR models. When evaluated in the acquired and intrinsic resistance models, n, tumor growth was blocked by 70% and 100%, respectively after treatment with ARV-110. ARV-110 has entered into a Phase 1 clinical trial and the preliminary data showed satisfactory safety and tolerability in patients. Therefore, ARV-110 will be promising treating strategy for patients with metastatic castration-resistant prostate cancer (CRPC) who have received standard care therapies.

### BCL2

BCL2 is the key member of the BCL2 family and regulates cell death (apoptosis) through inducing (pro-apoptotic) it or inhibiting it (anti-apoptotic). BCL2 is classified as an oncogene due to its important role as anti-apoptotic proteins.^127^ Different cancers will be caused when BCL2 is dysregulated, including lung cancers and lymphomas. Piers Blombery and coworkers found the emergence of a novel venetoclax resistant mutation (BCL2 F104I) in follicular lymphoma. Moreover, it was difficult to target BCL2 due to the protein–protein interaction (PPI) with Bcl-xl. Therefore, PROTACs will provide the promise for developing novel BCL2 inhibitors conquering the PPI and drug resistance.

**Fig. 8:**
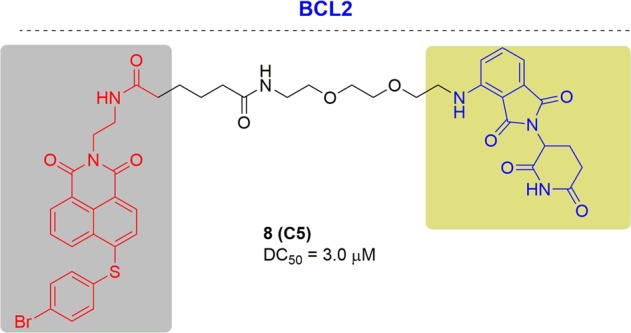
Representative PROTAC targeting BCL2.

The first BCL2 degrade was reported by Zhang and coworkers in 2019. the designed PROTACs for α-helix-mediated PPI targets to degrade BCL2^128^ (Fig. 8). The most potent and selective PROTAC, C5, degraded BCL2 with a DC_50_ of 3.0 μM and demonstrated cellular proliferation inhibition driven by the degradation efficiency.

Despite the development of BCL2 degraders, there have been no documents of effective PROTACs degrading drug-resistant BCL2.^129,130^

### BCL6

The transcriptional factor B-cell lymphoma 6 (BCL6) is a member of the bric-a-brac, tramtrack, broad complex/poxvirus zinc finger (BTB/POZ) family. It interacts with three corepressors (i.e., BCoR, SMRT, and NCoR) and possesses BTB, RD2, and zinc finger domains.^131^ BCL6 is required for germinal center B-cell formation and T lymphocyte differentiation.^132,133^ In addition, it has also been found to be involved in the differentiation and proliferation of diffuse large B-cell lymphomas (DLBCL) and follicular lymphoma cancers through a number of genetic alterations.^134,135^ Thus, BCL6 has been regarded as an effective therapeutic target for the therapy of autoimmune diseases and cancers.^136^ Although the current reported promiscuous^137^ and peptidomimetic^138^ based BCL6 inhibitors showed an attractive effect in vitro, they did not fulfill the promise of their preclinical data. A potent and selective tool is urgent for understanding BCL6 in human diseases.

**Fig. 9:**
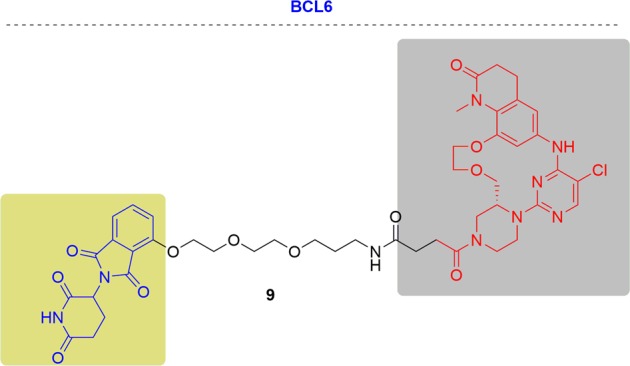
Representative PROTAC of BCL6.

In 2018, AstraZeneca first reported a potent and selective BCL6-targeting PROTAC, PROTAC **9**, by conjugating a designed BCL6 ligand and the CRBN ligand thalidomide (Fig. 9).^139^ PROTAC **9** showed dose-dependent degradation of BCL6 in all subcellular fractions but this degradation was not complete. Furthermore, PROTAC9 did not induce the phenotypic response in OCI-Ly1 and SUDHL4 cells for 16 day study.

### BCR-ABL

The fusion gene BCR-ABL is the main cause of chronic myelogenous lymphoma (CML).^140^ When the chromosomal translocation of the ABL gene from chromosome 9 to the BCR gene on chromosome 22, BCR-ABL is generated. BCR-ABL led to proliferation disorder of CML cells in patients by activating downstream signaling.^141^ The current focus on discovering novel drugs against the ABL tyrosine kinase of BCR-ABL for treating CML are still ATP-competitive inhibitors. Therefore, scientists have developed several BCR-ABL tyrosine kinase inhibitors and FDA have approved them to treat CML. Whereas, point mutations in the tyrosine kinase domain of BCR-ABL have been observed in a number of patients during the administration of kinase inhibitors and developed drug resistance eventually. therefore, three generations of inhibitors have been identified to address the growing drug resistance, including a first-generation TKI, imatinib;^142^ second-generation TKIs, dasatinib^143^ and nilotinib;^144^ and a third-generation TKI, ponatinib.^145^

**Fig. 10:**
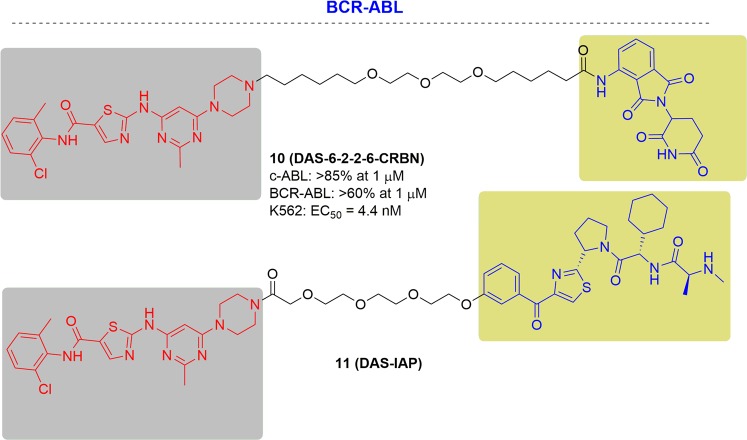
Representative PROTACs targeting drug-resistant BCR-ABL.

The first degrader of BCR-ABL was developed by Crews and coworkers in 2015. Based on bosutinib and dasatinib, BCR-ABL PROTAC was constructed that induced the degradation of c-ABL and BCR-ABL in the presence of either CRBN or VHL E3 ubiquitin ligase^146^ (Fig. 10). After evaluation, the dasatinib-derived PROTAC (DAS-VHL) mediated a clear (>65%) decrease of c-ABL at 1 µM. The dasatinib-CRBN (DAS-CRBN) PROTAC caused both degradation of c-ABL (>85% at 1 µM) and BCR-ABL (>60% at 1 µM). The dasatinib-derived BCR-ABL degrader caused cellular growth inhibition against BCR-ABL driven K562 with a half-maximal response concentration (EC_50_) of 4.4 nM. These degraders shed light on developing PROTACs treating drug-resistant BCR-ABL related disease.

In 2017, Naito and his colleagues reported the second BCR-ABL PROTAC deriving from dasatinib^147^ (Fig. 10). Subsequently, a new potent BCR-ABL degrader, named DAS-IAP, was developed by this group. DAS-IAP disclosed comparable activity in inhibiting CML cell growth and sustained anti-proliferative effects even when the drug was removed after short-term treatment. These results indicated that BCR-ABL degraders show more sustained inhibition of CML cell growth than ABL kinase inhibitors.

### BET

As the second highest cancer disease in men worldwide, prostate cancer has affected a number of people. Commonly, androgen deprivation for final remission is the most commonly used strategy to treat prostate cancer. Nevertheless, resistance to castration emerged. For those CRPC AR signaling blockers were the main treatments accompanied by a poor prognosis.^148^ However, secondary resistance invariably appeared, though CRPC had been treated by drugs targeting AR signaling.^149^ Recently, it has been proved that inhibition of the bromodomain and extraterminal (BET) family of proteins could disorder normal growth in preclinical models of CRPC.^150^ There have many reports about BET degraders^151–156^ and we will focus on the achievements of BET PROTACs that have overcome drug resistance.

**Fig. 11:**
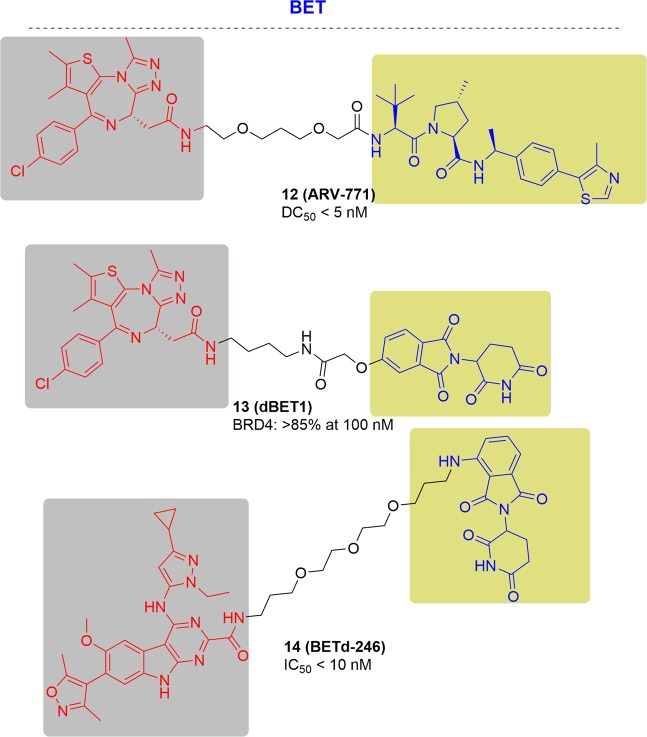
Representative PROTACs targeting drug-resistant BET.

In 2016, ARV-771 was illustrated by Crews and coworkers with a DC_50_ < 5 nM in 22Rv1 cells (Fig. 11) as a pan-BET degrader. ARV-771 caused degradation of c-MYC with an IC_50_ < 1 nM and apoptosis of cells through PARP cleavage.^152^ A VCaP tumor model which represents the clinical setting of AR overexpression following androgen-deprivation therapy, was chosen for evaluation of the potency of ARV-771 in vivo in the VCaP tumor model was chosen to. After treatment with ARV-771, the tumor growth inhibition was induced by 60% without significant loss in body weight. on the contrary, no tumor growth inhibition was observed in the enzalutamide-treated group. While, ARV-771 disclosed stronger efficacy and advantages in the aspect of treating CRPC compared to enzalutamide.

In 2015, the Bradner group reported another well-known BET degrader^151^ (Fig. 11). The conjugation of JQ1 and pomalidomide afforded dBET1. Treating MV4-11 cells with dBET1 led to a significant loss of BRD4 (>85%), which was achieved with concentrations as low as 100 nM after 18 h of treatment, and dBET1 pronounced a potent and superior proliferation inhibition of MV4-11 cell at 24 h compared to JQ1. Moreover, dBET1 was able to degrade BRD4 and inhibit tumor growth in vivo in a murine hind-limb xenograft model with human MV4-11 leukemia cells without affecting the animal weight and normal complete blood counts after the degrader treatment. More significant downregulation of MYC was observed when compared to vehicle group in excised tumors.

For triple-negative breast cancer (TNBC) patients, chemotherapy can generally give a high response.^153^ However, the residual tumors cause high rates of metastatic disease due to the amplification of MCL1 loci, which was one of the most common genetic changes in chemo-refractory tumors. Therefore, scientists have proven that MCL1 is the fusion of both an intrinsic and acquired resistance factors in TNBC patients. Resistance has limited the efficacy of a variety of anticancer agents. In 2018, the Wang group reported BETd-246 derived from their BET inhibitor (BETi-211) to degrade BET proteins for treating TNBC (Fig. 11). BETd-246 induced degradation of BRD2, BRD3 and BRD4 in a dose-dependent manner. After treatment for 1 h or 3 h with 30–100 nmol/L of BETd-246 or with 10–30 nmol/L of BETd-246 respectively, the proteins BRD2-4 were nearly completely depleted. BETd-246 inhibited the TNBC cell growth with an IC_50_ < 10 nmol/ the and led to rapid and time-dependent downregulation of MCL1 protein in the tested TNBC cell lines. Treating a patient-derived xenograft (PDX) model of TNBC with BETd-246, a dose of 5 mpk, i.v., three times per week for 3 weeks, effective anti-tumor activity was observed, similar as the inhibitory effects of BETi-211 at 50 mpk, daily, after oral dosing, 5 days a week for 3 weeks. This finding suggested a promising approach to target MCL1 for TNBC to overcome clinical resistance.

### BRD9 and BRD7

BRD9 is the bromodomain-containing subunit of the BAF (BRG-/BRM-associated factor)^157^ and its close homolog BRD7 is the subunit of PBAF (polybromo-associated BAF).^158^ BAF and PBAF are two variants of the SWI/SNF complex, which regulate gene expression, DNA replication and DNA repair.^159^ Overexpression of BRD9 is found in several cancers, including cervical cancer.^160^

**Fig. 12:**
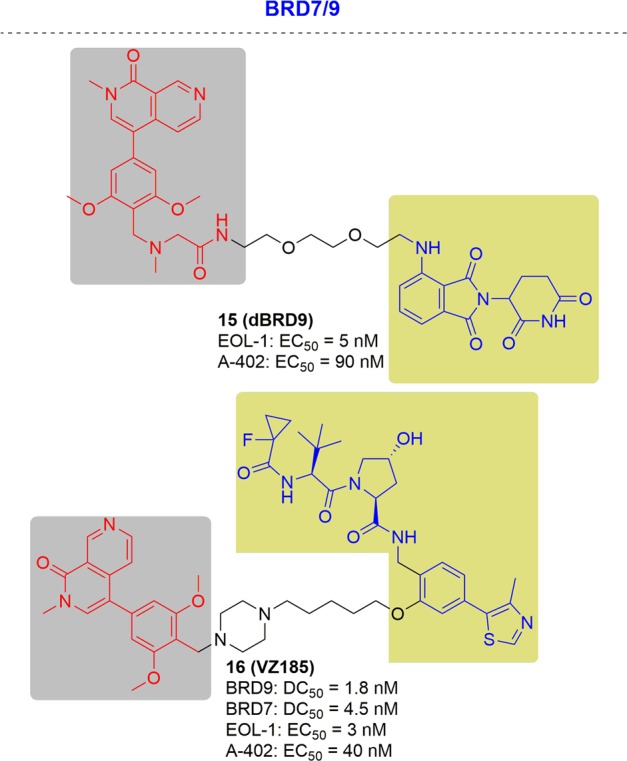
Representative PROTACs targeting BRD9/7.

In 2017, the Bradner group designed and characterized the first degrader of BRD9, which showed obvious degradation of BRD9 at 50 nM^161^ (Fig. 12). The anti-proliferative effect of dBRD9 was slightly better than that of an inhibitor in the human AML MOLM-13 cell line.

In 2019, Ciulli and coworkers described a degrader by conjugating the ligands of VHL and BRD9. the reported degrader could degrade BRD9 and BRD 7 with DC_50_ values of 1.8 and 4.5 nM respectively^162^ (Fig. 12). Two cell lines, EOL-1 (acute myeloid eosinophilic leukemia) and A-204 (malignant rhabdoid tumor), which are sensitive to BRD9 inhibition/degradation and dependent on an active BAF complex, were selected to study the impact of degrader-induced BRD7/9 degradation on the viability of cancer cells Metabolically active cells was referred as those cells with the presence of the cellular ATP presence. The CRBN-based degrader dBRD9 showed cytotoxic effects in both cell line, with EC_50_ values of 5 nM (EOL- 1) and 90 nM (A-402) and proved to be equipotent to VZ185 with EC_50_ values of 3 and 40 nM, respectively.

### BTK

B-cell receptor (BCR) is an important regulator in B cell signaling in adhesion, survival, and growth. For BCR pathway, BTK is indispensable since it worked as a membrane proximal signal molecule for the activation and proliferation of B cell.^163^ Presently, ibrutinib has been approved for treating MCL and activated B-cell-like (ABC)-DLBCL by covalent binding.^164^ However, MCL patients have developed drug resistance after receiving ibrutinib treatment due to the missense BTK mutation of C481S.^165^ Ibrutinib also lost the inhibitory efficacy of DLBCL tumor cell growth resulting from the BTK C481S mutant.

**Fig. 13:**
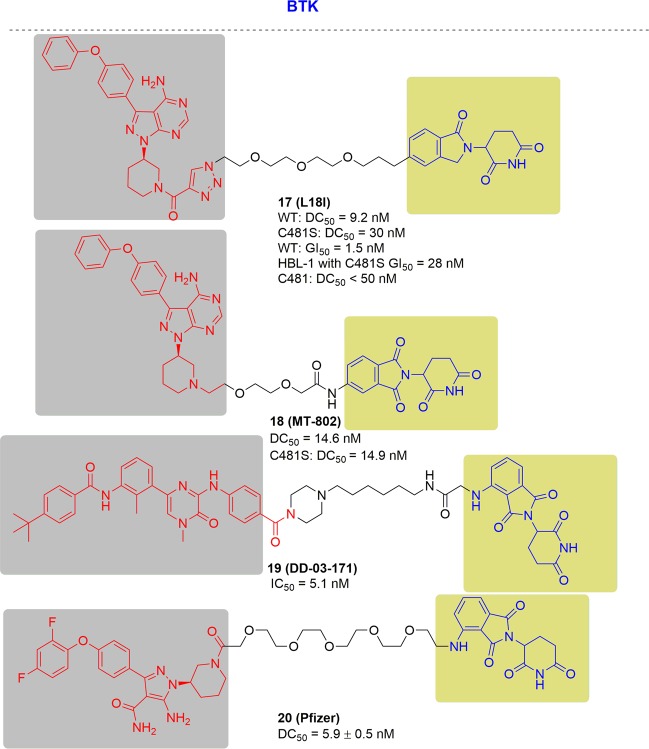
Representative PROTACs targeting drug-resistant BTK.

In 2018 and 2019, Rao and coworkers first reported two panel of novel BTK degraders for knockdown of drug-resistant BTK^166,167^ (Fig. 13). At first, the potent degrader P13I showed high efficiency of degrading both the wild type and ibrutinib-resistant C481S BTK, with DC_50_ at 9.2 and 30 nM respectively. In addition, P13I afforded slightly better growth inhibition with GI_50_ (50% growth inhibition concentration) values of 1.5 nM when compared to ibrutinib in the wild-type BTK cells. Moreover, P13I effectively downregulated the self-phosphorylation of C481S mutant BTK at low concentration and while ibrutinib failed. Hence, P13I could significantly inhibit the growth of HBL-1 cells expressing BTK C481S mutant with a GI_50_ values of approximately 28 nM. While ibrutinib lost inhibitory efficacy in the mutant BTK cells. After that, further optimized BTK PROTACs with a great improvement in water solubility were generated. Among the second generation, L18I was the representative degrader, which had the ability to degrade different C481 BTK mutants with DC_50_ values lower than 50 nM. Moreover, L18I could afford rapid tumor regression in mouse xenograft models inoculated with C481S BTK HBL-1 cells with a 30 or 100 mpk dose, and the tumor reduced by 36% and 63% respectively. In contrast, the mice administered ibrutinib performed serious tumor burden. The above results suggest that the BTK-targeting PROTAC degraders provided great potential of inhibiting the BTK functions especially for ibrutinib-resistant lymphomas.

At almost the same time, the Crews group reported another BTK PROTAC, MT-802, derived from ibrutinib^168^ (Fig. 13). For wild-type BTK, MT-802 caused BTK degradation efficiently with a DC_50_ of 14.6 nM, with maximal degradation at 250 nM. MT-802 retained the same potency against C481S BTK with a DC_50_ of 14.9 nM. In addition, MT-802 was capable of reducing the phosphorylation of BTK in cells isolated from CLL patients with the C481S mutation while ibrutinib could not.

In 2018, a multikinase degrader that combined a highly promiscuous kinase inhibitor with a cereblon-binding ligand was designed by Nathanael S. Gray, and this multikinase degrader could degrade several kinases, including BTK^169^ (Fig. 13). In 2019, a more specific BTK degrader named DD-04-015 was released, which effectively and selectively degraded BTK. Treatment with DD-04-015 for 4 h led to efficient degradation at 100 nM. In addition, DD-04-015 exhibited a similar cell proliferation effect compared to RN486 in TMD8 cells after 3 days of treatment. With further optimization, lead compound DD-03-171 with the ability to degrade C481S-BTK was developed. DD-03-171 exhibited stronger antiproliferation inhibition of mantle cell lymphoma (MCL) cells in vitro with an IC_50_ of 5.1 nM and efficient anti-cancer effects on PDX in viv*o*.

Pfizer also disclosed PROTACs targeting BTK in 2018, derived from a previously reported covalent phenyl-pyrazole to bind BTK and pomalidomide to bind CRBN^170^ (Fig. 13). The most potent BTK degrader led to efficient degadation of BTK with a DC_50_ of 5.9 ± 0.5 nM after 24 h of treatment in Ramos cells. When evaluated in vivo, efficient BTK degradation was also observed in the lung and spleen in the BTK degrader-treated rats. This BTK PROTAC applied to BTK mutants was not revealed in the report.

### CDK4/6

**Fig. 14:**
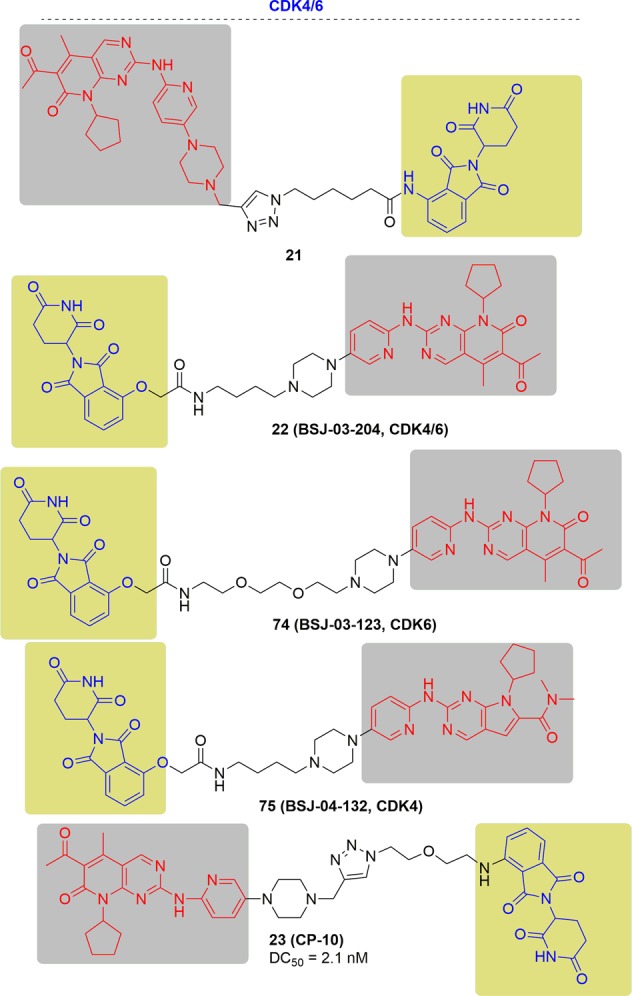
Representative PROTACs targeting CDK4/6.

In 2019, Burgess and colleagues reported their work on the development of dual CDK4/6 degraders^171^ (Fig. 14). The developed PROTACs could degrade CDK4/6 with DC_50_ values ranging from 20 to 50 nM and inhibit the cell growth in an admissible level. However, their compounds did not show the efficacy in cells overexpressing CDK4/6.

In 2019, Gray and coworkers varied the linkers of the bifunctional molecules to find dual CDK4/6 degraders (BSJ-03-204) and selective CDK4 and CDK6 degraders (BSJ-04-132 or BSJ-03-123, respectively)^172,173^ (Fig. 14). These degraders could degrade target proteins at 100 nM and revealed better anti-proliferative effects in comparison to CDK4/6 inhibitors. Moreover, in Granta-519 cells that was characterized by overexpression of cyclin D1. the degrader BSJ-02-162 or BSJ-03-204 could result in, marked degradation of CDK4/6and induced G1 cell cycle arrest at the same time.

The most potent degrader was reported by Rao and coworkers, which was derived from pomalidomide and palbociclib, and showed specific and remarkable potency on CDK6 degradation with a DC_50_ of 2.1 nM^174^ (Fig. 14). Moreover, the PROTACs still held strong degradation and proliferation through the inhibition of hematopoietic cancer cells with copy-amplified/mutated forms of CDK6.

Although several PROTACs targeting CDK4/6 have been reported, but how to develop the PROTACs applied for CDK inhibitor-resistant cells is until challenging now.^175,176^

### CDK8

Cyclin-dependent kinase 8 (CDK8) is a member of the cyclin-dependent kinase family that plays an important role in promoting cell cycle phase transition, initiating DNA synthesis, and regulating cell transcription during cell proliferation and differentiation, especially in oncogenic signaling pathways, including the TGF-β signaling pathway, the Wnt-β-catenin pathway, the p53 pathway, and the serum and hypoxia response network.^177–179^ The study found that overexpression of the CDK8 gene disrupted cell proliferation, differentiation and apoptosis, which could accelerate the growth and division of cancer cells, such as cervical cancer, colorectal cancer, gastric cancer, malignant melanoma, and so on.^180^ Although CDK8 inhibitors have been used and have gradually received increasing attention, their effectiveness in the treatment of various cancers has not yet been confirmed.^181^ Therefore, the development of PROTACs for degrading the protein CDK8 has become a new strategy to overcome these shortcomings.

**Fig. 15:**
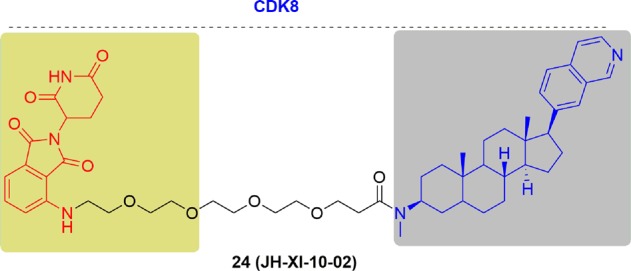
Chemical structure of the reported CDK8 PROTAC.

Nathanael S. Gray and coworkers first synthesized a series of compounds based on cortistatin A. The results showed that the designed derivatives had slightly reduced biological activity toward CDK8 in vitro and cellular assays. Then, based on this scaffold, they designed JH-XI-10-02 (**24**),^182^ a potent degrader of CDK8 (Fig. 15). They observed significant degradation of CDK8 after treatment with **24** at 1 μM for 24 h in Jurkat cells. Then, they verified the mechanism by which degradation was mediated via CRBN by using the negative control in CRBN knockout Molt14 cells. The development of CDK8 degraders not only provided a tool for regulating CDK8 protein levels in vivo, but also offered an effective strategy for treating cancer with CDK8 degraders.

### CDK 9

Cyclin-dependent kinase 9 (CDK9) is a member of the cyclin-dependent protein kinase (CDK) family, which can form the subunit of the positive transcription elongation factor b (P-TEFb) complex with cyclin T and plays a critical role in the transcriptional elongation of a number of oncogenes.^183–185^ It is ubiquitously expressed in all tissues and a variety of malignancies.^186^ Preclinical studies exhibited that selectively targeting CDK9 may have therapeutic potential in cancer treatment and other human diseases.^187,188^ However, CDK9 shows a high level of conservation sequence with other CDK family members, which makes it challenging to develop selective CDK9 inhibitors.^189^ Because of the different surface shape and the different distribution of lysine residues on CDKs surface, this would provide a unique opportunity to develop a selective CDK9-targeting PROTAC, which requires an appropriately exposed lysine residue surface for ubiquitination and proteasome degradation.^190^

**Fig. 16:**
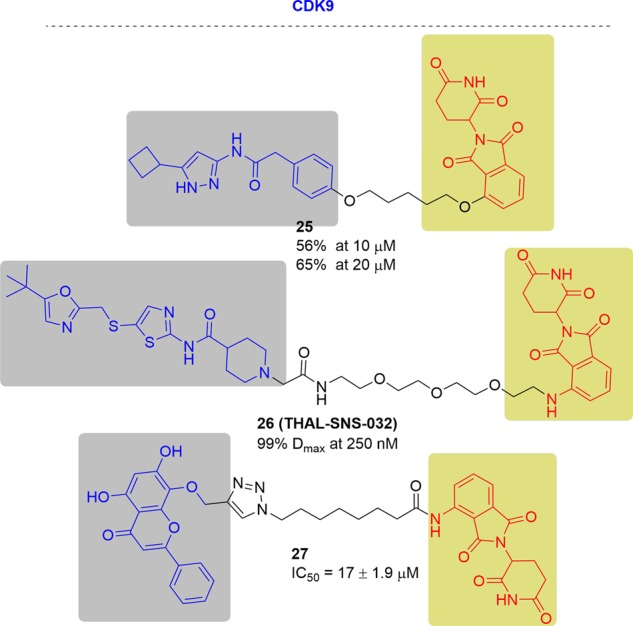
Representative PROTACs of CDK9.

In 2017, Sandeep Rana and his coworkers developed the first selective CDK9 degrader by conjugating aminopyrazole analog and the CRBN ligand thalidomide^191^ (Fig. 16). In HCT116 cells, the western blots showed that the CDK9 degrader can reduce approximately 56 and 65% of CDK9 protein at 10 and 20 µM, respectively, sparing other CDK family members.

In 2018, the Nathanael S. Gray group developed a selective CDK9 degrader, THAL-SNS-032, which consisted of the CDK multitargeting kinase inhibitor SNS-032 and a thalidomide derivative^192^ (Fig. 16). THAL-SNS-032 induced the rapid degradation of CDK9 with a 99% *D*_max_ at 250 nM in MOLT 4 cells after 6 h of treatment, but it did not affect the levels of other SNS-032 targets. In addition, THAL-SNS-032 showed a longer pharmacodynamic effect than inhibitors.

In contrast to the aminopyrazole and aminothiazole scaffold-based CDK9-targeting PROTACs from the above two groups,^191,192^ Zhiyu Li and coworkers produced the CDK9 degrader **27** by conjugation of the natural product wogonin to pomalidomide^193^ (Fig. 16). PROTAC **27** selectively degraded CDK9 and showed more potent cell proliferation inhibition activity (IC_50_ = 17 ± 1.9 μM) than wogonin (IC_50_ = 30 ± 3.5 μM) in MCF7 cells. In addition, **27** was much less active against the cell lines with low levels of CDK9 expression, such as L02 (IC_50_>100 μM).

### CK2

Casein kinase 2 (CK2) is an omnipresent,and constitutively active serine/threonine protein kinase with different kinds of functions.^194^ Overexpression of CK2 is relevant to occurrence of cancers.^195^

**Fig. 17:**
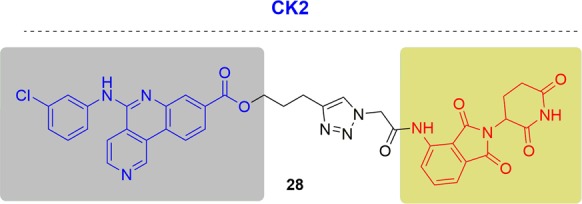
Representative PROTAC targeting CK2.

In 2018, Gou and coworkers reported PROTACs targeting CK2 by conjugating a CK2 inhibitor (CX-4945) and pomalidomide^196^ (Fig. 17). Among the reported degraders, compound **28** showed CK2 degradation in a dose- and time-dependent way. When CK2 was degraded, reduced phosphorylation of Akt and the upregulation of p53 was observed. Surprisingly, the degrader **28** showed a similar cytotoxicity to CX-4945 with the CK2 inhibitor, while the mechanism was quite different. The PROTACs targeting CK2 proteins seem to be a potential strategy for cancer treatment.

### c-Met

c-Met is a transmembrane-spanning RTK and the receptor of hepatocyte growth factor (HGF), which is also known as scatter factor (SF). c-Met and HGF have causal roles in cancer cell survival, growth, angiogenesis and metastasis. Once bound to HGF/SF, c-Met dimerizes, and transphosphorylation happens in the kinase domain (Y1234 and Y1235) and C-terminal docking domain (Y1313, Y1349, Y1356, and Y1365).^197^ The docking domain recognizes many downstream cellular effectors, including Src, Gab1, Crk, Grb2, SHC, and PI3K, which play important roles in cancer biology. Inhibitors of c-Met kinase have been developed in the past near 20 years, but they have been disappointing in clinical trials. This suggested that a kinase-independent function might drive oncogenesis and degradation and might be a potential advantage over inhibition.

**Fig. 18:**
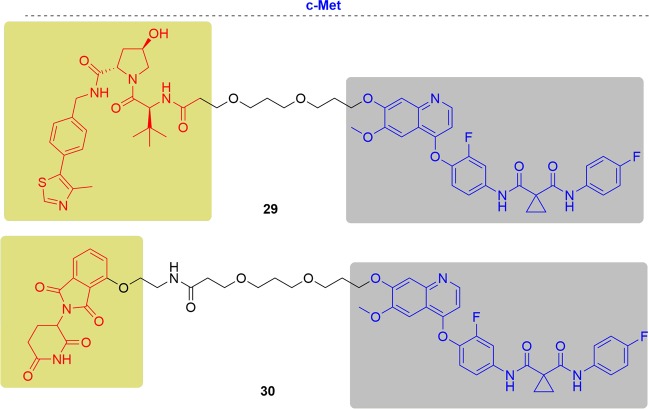
Representative PROTACs of c-Met.

Therefore, the Crews group developed the c-Met-targeting PROTAC based on the promiscuous inhibitor foretinib^47,103,198^ (Fig. 18). Both VHL and CRBN PROTACs could induce the degradation of c-Met in a dose- and time-dependent manner. The rapid clearance of c-Met by the foretinib-based VHL PROTAC was observed within 6 h, which provided an advantage over RNAi. RNAi usually requires transfection reagents or exogenous selection pressure which can affect other biological processes. Because RTKs are also degraded by HSP90, they found that the foretinib-based VHL PROTAC and the HSP90 inhibitor 17-AAG had additive effects on c-Met degradation. They also confirmed that the foretinib-based VHL PROTAC induced internalization of c-Met from the cell surface by confocal immunofluorescence microscopy. Exon 14-deletion c-Met lacks the juxta membrane domain recruitment site (Y1003) for its endogenous E3 ligase and thus the natural “off-switch” for HGF-induced signaling is no longer present. Foretinib-based VHL PROTAC could induce the degradation of the exon 14-deletion c-Met despite not being degraded by the natural mechanism, which provided another example that degradation might be advantageous over inhibition.

### DHODH

Dihydroorotate dehydrogenase (DHODH) is a flavin mononucleotide (FMN)-enzyme in mitochondria that catalyzes the oxidation of dihydroorotate to orotate with coenzyme Q as a cofactor in the de novo biosynthesis of pyrimidine. It provides building blocks for further synthesis of RNA, DNA, glycoproteins, and phospholipids.^199^ The inhibition of DHODH’s activity has been proposed as a promising therapeutic strategy for viral infection, cancer, arthritis, and immunosuppression. For example, brequinar, a strong DHODH inhibitor, has attracted much attention in previous studies but has failed in clinical studies due to its side effects and poor solubility.

**Fig. 19:**
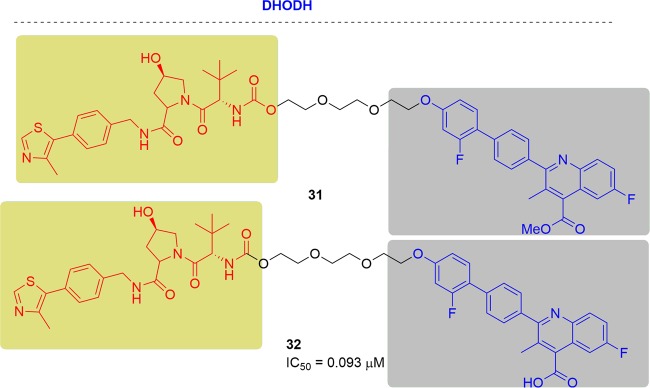
Representative PROTACs of DHODH.

The Neamati group designed PROTAC probes based on brequinar to better understand the therapeutic relevance of DHODH in cancer^200^ (Fig. 19). Probe **32** contained the crucial carboxylic acid and maintained excellent potency in an enzymatic assay (IC_50_ = 0.093 μM). In contrast, methyl ester **31** did not inhibit the activity of DHODH (IC_50_ > 200 μM). However, **32** didn’t inhibit cell growth in DHODH-sensitive HCT-116 cells. Conversely, **31**was more potent in HCT-116 cells, which may be a result of superior cellular permeability. Moreover, **31** hindered new colony formation better than brequinar, which suggested a more in-depth biological property **31**. Unfortunately, any protein degradations were not observed with both **31** and **32**, because of the possible significantly different protein ubiquitination system in mitochondria.

### EGFR and HER2

Epidermal growth factor receptor (EGFR) is a glycoprotein with tyrosine kinase activity that is a major member of the erythroblastosis oncogene B (ErbB) family.^201^ The EGFR family contains four subtypes: EGFR (ErbB1, HER1), ErbB2 (HER2), ErbB3 (HER3), and ErbB4 (HER4).^202^ EGFR is involved in tumor cell proliferation, angiogenesis, tumor invasion, metastasis, and inhibition of apoptosis. The overexpression of EGFR plays an important role in the progression of malignant tumors, such as glioblastoma, NSCLC, head and neck cancer, breast cancer, colorectal cancer, ovarian cancer, prostate cancer, pancreatic cancer and so on.^201,203,204^ EGFR-targeted therapy had led to the development of many excellent EGFR inhibitors with high selectivity and few side effects.^49,146,205^ However, there is still drug resistance and a low clinical response rate, caused by new mutations in prolonged clinical medication.^62,63,206^

**Table:** 

Cell line	Mutation	PROTAC	Warhead	DC_50_ (nM)	*D*_max_ (%)
OVCAR8	Wild-type	**33**	lapatinib	39.2	97.6
HeLa (overexpressed mutant)	Exon 20 ins	**33**	lapatinib	736.2	68.8
HCC827	Exon 19 del	**34**	gefitinib	11.7	98.9
H3255	L858R	**34**	gefitinib	22.3	96.6
H1975	L858R/T790M	**35**	afatinib	215.8	79.1

**Fig. 20:**
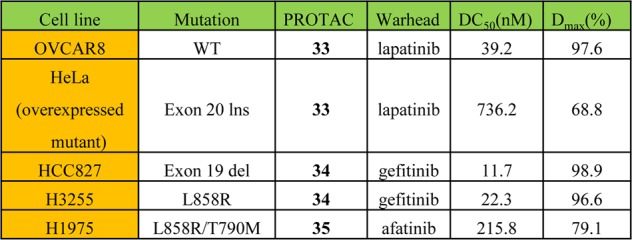
Potency of EGFR PROTACs in different cell lines.^198^

**Fig. 21:**
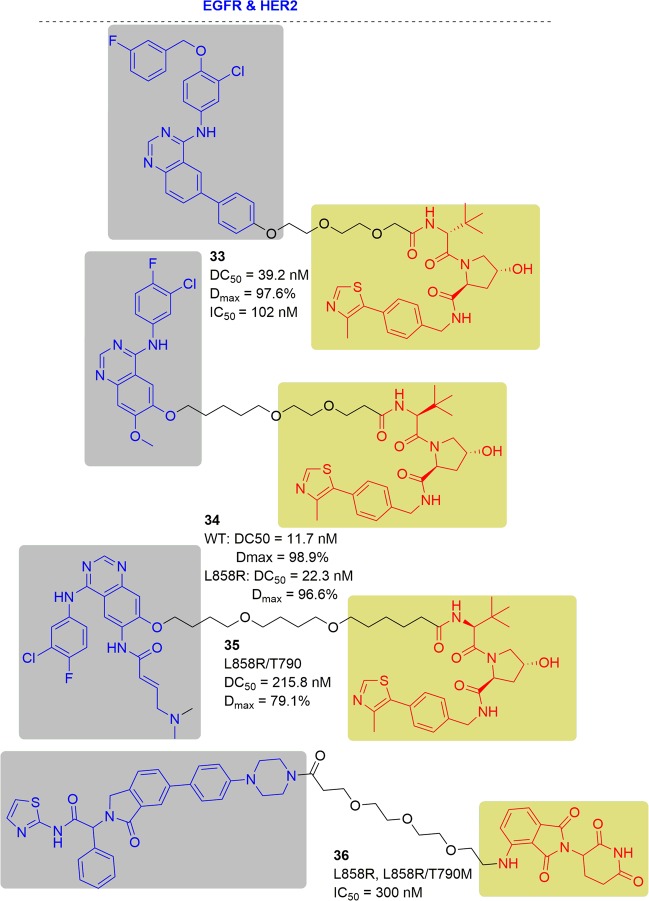
Representative PROTACs of EGFR and HER2.

Craig M. Crews and coworkers reported some EGFR degraders based on the kinase inhibitor lapatinib, mutant-EGFR selective gefitinib, the second-generation inhibitor afatinib and a VHL ligand (Figs. 20 and 21). They found that all the degraders were capable of inducing EGFR degradation.^198^ For example, compound **33** induced EGFR degradation with a DC_50_ = 39.2 nM and a *D*_max_ = 97.6% in the OVCAR8 cell line. Compound **33** had greater anti-proliferative efficacy with an IC_50_ = 102 nM in SKBr3 cells. Moreover, the results showed that compound **33** also could degrade exon-20 insertion mutant form of EGFR in the HeLa cell line. Mutant-EGFR selective gefitinib was used to replace the warhead to develop the compound **34**, which enabled the degradation of exon-19 deletion EGFR (DC_50_ = 11.7 nM and *D*_max_ = 98.9%) in the HCC827 cell line and the L858R activating point mutation (DC_50_ = 22.3 nM and *D*_max_ = 96.6%) in the H3255 cell line. When the second-generation inhibitor afatinib was employed to develop compound **35**, it could degrade gefitinib-resistant double mutant (L858R/T790M) EGFR with DC_50_ = 215.8 nM and D_max_ = 79.1% in the H1975 cell line.

Considering that lapatinib is also an effective binder to other RTKs, they evaluated the potential degradation ability of compound **33** toward HER2. They found that compound **33** was able to induce HER2 degradation at 25 nM, but it showed no selectivity between EGFR and HER2. Based on the results, they designed new compounds with different linkers and tested the degradation activity and selectivity to HER2. Finally, these compound only selectively degraded EGFR and had no effect on HER2.

In addition, there was patent focused on the design of EGFR degraders. In the patent, they found that compound **36** had good EGFR degradation activity. It is obvious that EGFR degradation could be observed with compound **36** at 100 nM. Compound **36** also had good antiproliferative activity against the L858R mutant and L858R/T790M mutant with an IC_50_ of 300 nM in the Ba/F3 cells.

### eIF4E

Eukaryotic translation initiation factor 4E (eIF4E) is a cap-binding protein that specifically recognizes the m7GpppX cap at the 5′ terminus of coding mRNAs, which affected the initiation of eukaryotic translation.^207–209^

Binding of eIF4E with the mRNA cap results in the recruitment of translational machinery, then initiates protein synthesis at the transcript’s start codon. eIF4E has great influence on cell proliferation, differentiation and metastasis.^210^ Studies have shown that eIF4E is overexpressed in many malignant cell lines and primary tumors in animals and humans, including breast cancer, lung cancer, and non-Hodgkin’s lymphomas among others.^211^ It has been reported that inhibition of eIF4E function can slow tumor growth and induce apoptosis.^212^ Therefore, the development of novel eIF4E degraders is a new strategy for the treatment of many cancers.^213,214^

**Fig. 22:**
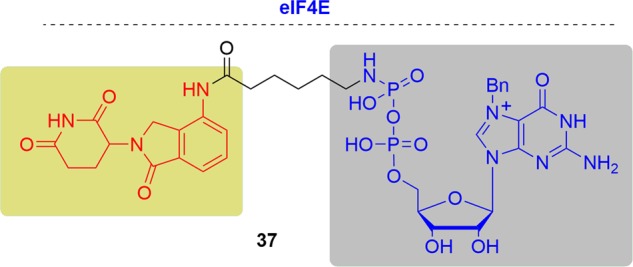
Representative PROTAC of eIF4E.

Amanda L. Garner and coworkers first developed novel PROTACs for eIF4E degradation (Fig. 22). They generated a small library of GxP (GMP or GDP) derivatives conjugated to lenalidomide and VHL ligand.^215^ The previously report has shown that GxP (GMP or GDP) was good inhibitor of eIF4E so which was chosen as the Bn7GxP scaffold. To test the degradation ability of the compounds they developed a cap competition assay in vitro. In this assay, HEK293 cell lysates were incubated with m7GxP (GMP or GDP) agarose resin to enable the affinity purification of eIF4E.

In the biochemical characterization tests, they found that all the GDP conjugates were active, while the GMP derivatives had no degradation ability. Especially, compound **37** was able to significantly degrad eIF4E at 50 μM, and eIF4E was completely degraded when the compound concentration increased to 500 μM. However, when MDA-MB231 and K562 cells were treated with these compounds, no intracellular degradation of eIF4E was observed despite concentrations of the compound up to 500 μM. They hypothesize that the main reason was the low cellular permeability, as has been observed with other cap analogs.

### ER

ER is a regulator of gene expression and many biological processes as a nuclear receptor including ERα and ERβ. Eighty percent of all newly diagnosed cases of breast cancer are ERα positive,^194^ as ERα is considered the major regulator that transduces estrogen signaling in the female reproductive tract and mammary glands.^216^ The current treatment standard is fulvestrant which acts through selectively degrading the estrogen receptor for ER+ metastatic breast cancer. Although fulvestrant has realized therapeutic intervention by the degradation of ER, up to 50% of the ER remains in comparison with baseline levels after six months of treatment with fulvestrant. Consequently, drug resistance has emerged for a variety of ER+ breast cancers, although approved treatments have provided success in this patient population.

In 2018, ARV-471 was documented by Arvinas as an ER degrader for treating ER+ metastatic breast cancer as an oral therapy (Fig. 22). ARV-471 induced obvious degradation of ER at 11 nM in a variety of breast cancer cell lines. ARV-471 displayed a 99% inhibitory effect on tumor growth at 10 mpk and 106% effect at 30 mpk (below) in the PDX from an ESR1 mutant patient model after oral administration. Meanwhile, fulvestrant demonstrated less potent inhibition of tumor growth. This data illustrated that ARV-471 exhibited much potent inhibition of tumor growth compared to fulvestrant. As released by Arvinas, ARV-471 held promising activity and potency as both a single agent and as a combination therapy with CDK4/6 inhibitors through degrading the ER. A Phase 1 study in Q3 was initiated in 2019 by Arvinas for women with locally advanced or metastatic ER+ positive/HER2- negative breast cancer.

**Fig. 23:**
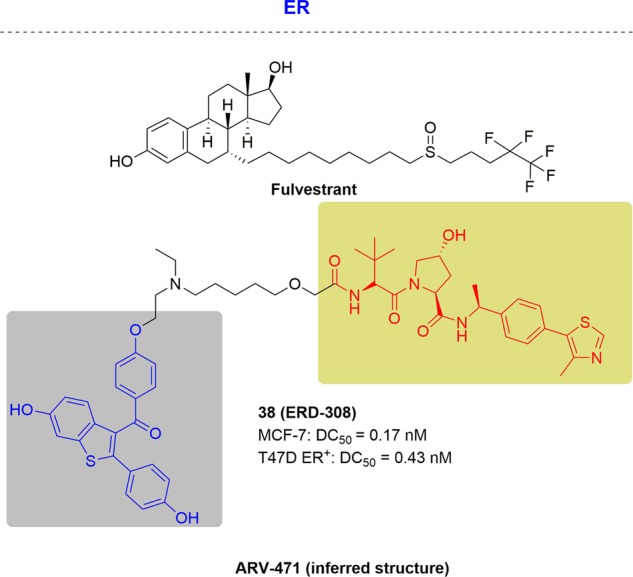
Representative PROTACs targeting drug-resistant ER.

In 2018, Wang and his colleagues disclosed a highly potent ER degrader called ERD-308^217^ (Fig. 23). ERD-308 induced efficient ER degradation in MCF7 and T47D ER+ breast cancer cell lines, with DC_50_ of 0.17 and 0.43 nM respectively. By comparison with fulvestrant, degrader caused more complete target degradation, and demonstrated stronger inhibition of cell growth in MCF-7 cells. These data demonstrate a new kind of ER degraders for the treatment of advanced and metastatic ER+ breast cancer.

### ERK1 and ERK2

ERK1 and ERK2 are closely related serine/threonine kinases and participate in the Ras-Raf-MEK-ERK signal transduction cascade, which is involved in many biological processes including cell adhesion, cell cycle progression, cell migration, cell survival, differentiation, metabolism, proliferation, and transcription by catalyzing the phosphorylation of hundreds of cytoplasmic and nuclear substrates.^218,219^ This signaling pathway is implicated in numerous cancers. Degradation of ERK1 and ERK2 levels could be an advantageous approach compared with inhibition since a significant proportion of signaling by ERK1 and ERK2 arises from protein–protein interactions in addition to the catalytic activities.

**Fig. 24:**
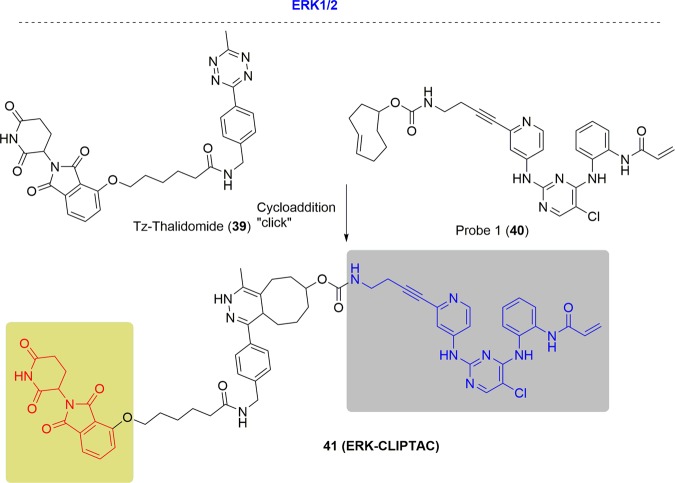
Representative PROTACs of ERK1 and ERK2.

The Heightman group proposed that PROTACs possess high molecular weight, which limits their cellular permeation, and other drug-like properties^62^ (Fig. 24). They designed two smaller precursors that could intracellularly form the ERK1 and ERK2-targeting PROTAC molecule by a bio-orthogonal click combination based on a covalent inhibitor and tetrazine-tagged thalidomide. The ERK1 and ERK2 degradation was complete after 16 h in the presence of probe **40** (10 μM) and Tz-Thalidomide **39** (10 μM). When ERK-CLIPTAC was prepared prior to addition to cells, there was no degradation of ERK1 or ERK2. These results indicated a lack of cell permeability of the PROTAC molecule and confirmed that the degradation resulted from the click formation of the PROTAC from the two smaller precursor molecules following to their entry into cells.

### ERRα

As a member of the orphan nuclear receptor superfamily, estrogen-related receptors (ERRs) play important roles in maintaining homeostasis in the body, including early development regulated by ERRβ and metabolic balance associated with ERRα and ERRγ.^220–222^ ERRα shares relatively high homology with estrogen receptor α (ERα) and is responsible for regulating metabolism and energy homeostasis by interacting with multiple transcriptional cofactors, such as the peroxisome proliferator-activated receptor γ coactivator 1 proteins (i.e., PGC-1α and PGC-1β), receptor-interacting protein 140 corepressor (RIP-140), etc.^223–225^

**Fig. 25:**
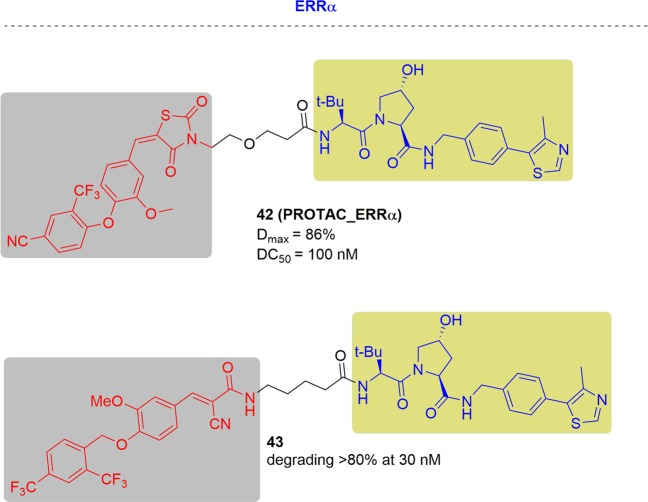
Representative PROTACs targeting ERRα.

In 2015, Crews and coworkers reported the first PROTAC (PROTAC_ERRα) to induce the degradation of ERRα^205^ (Fig. 25). The designed degrader exhibited a decrease in ERRα levels dose dependently in MCF-7 cells. The DC_50_ was about ~100 nM and the *D*_max_ was 86%. In addition, they evaluated the efficiency of the ERRα PROTAC in vivo. After treatment with the degraders, significant decreases in ERRα levels were observed in the hearts and kidneys and MDA-MB-231 tumors by approximately 44%, 44% and 39%, respectively when compared to the administration of an equal volume of ERRα inhibitors. The PROTAC_ERRα PROTAC retained its degradation activity in vivo by distributing into tissues and reducing ERRα levels upon target engagement.

In 2019, the Ding group developed a series of *(E)*-3-(4-((2,4-bis(trifluoromethyl)benzyl)oxy)-3-methoxyphenyl)-2-cyanoacrylamide derivatives to identify new estrogen-related receptor α (ERRα) degraders^226^ (Fig. 25). The representative degrader, **43**, was able to specifically degrade the ERRα protein by >80% of 30 nM, which represented as one of the most selective and potent ERRα degraders until now.

### FAK

Focal adhesion kinase (FAK or PTK2) is widely expressed in different species and has more than 90% homology in amino acid sequence.^227^ It exerts kinase-dependent enzyme function and kinase independent scaffold function, both of which are crucial in the development of cancer (e.g., invasion, metastasis, and angiogenesis), early embryonic, reproduction and so on.^228–231^ Except for the kinase domain,^232^ FAK contains three other functional domains: band 4.1, Ezrin, Radixin, Moesin (FERM) N-terminal domain, proline-rich regions (PRI-III), and focal adhesion targeting (FAT) C-terminal domain.^233,234^ The FERM domain plays an important role in cellular regulation.^235–237^ The PR and FAT domain mainly participate in different protein–protein interactions,^238^ all of which mediate FAK kinase independent signaling and participates in the formation of large signaling complexes.^239,240^ However, the current medicinal chemistry toolbox limits the development of chemical entities for FAK inhibition and ignores the FAK scaffolding functions. Although a few FAK inhibitors have been proven to be effective in preclinical studies, clinical success has yet to be observed.^239,241,242^ In addition, drug resistance may lead to by traditional kinase inhibitors, due to they can only act on kinase domain. Therefore, new strategies to eliminate both the FAK enzymatic functions and the scaffolding functions are very important for FAK-related diseases.

**Fig. 26:**
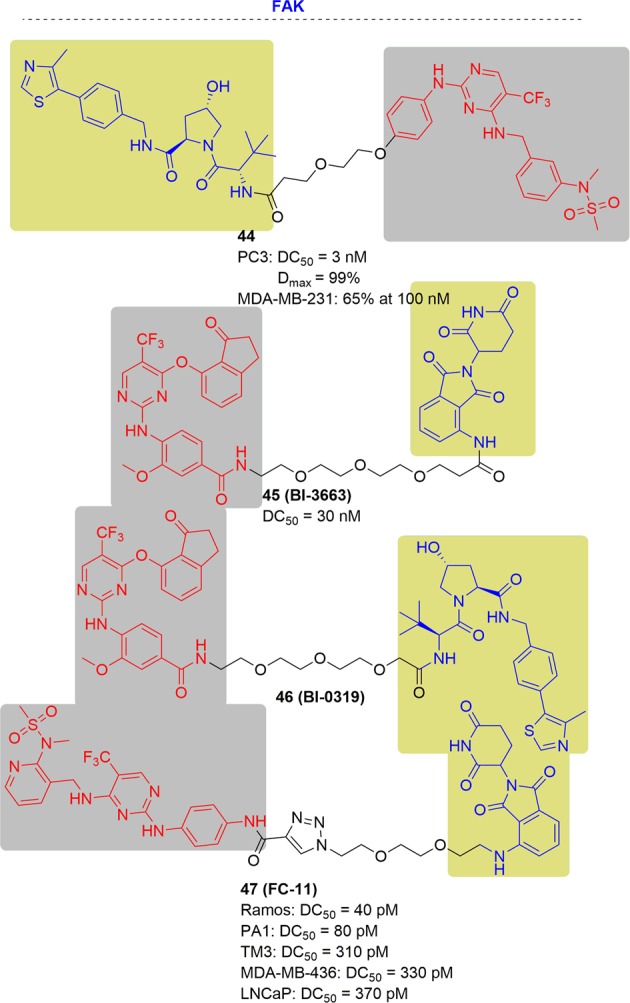
Representative PROTACs of FAK.

In 2018, the Craig M. Crews group reported the first nanomolar FAK-targeting PROTAC, **44**, based on defactinib and a VHL E3 ubiquitin ligase^100^ (Fig. 26). Compound **44** showed better protein selectivity and potent protein degradation. Its DC_50_ was 3 nM and D_max_ was 99% in serum-free treated PC3 cells at 24 h. In addition, **44** significantly impaired cell migration and resulted in a reduction of wound healing after treatment with 50 nM and 250 nM after 24 h of treatment in human triple negative breast cancer cells (MDA-MB-231). In a Transwell cell invasion assay, **44** reduced MDA-MB-231 cell invasions by as much as 65% at a concentration of 100 nM for 24 h. Furthermore, **44** also outperformed defactinib with respect to FAK activation and downstream signaling. However, it did not affect cell proliferation.

In 2019, the Peter Ettmayer group developed two highly selective and functional FAK-targeting PROTACs (BI-3663 and BI-0319) by utilizing both CRBN and VHL ligands^243^ (Fig. 26). BI-3663 (CRBN-based) degraded FAK with DC_50_ of 30 nM in a panel of 11 human hepatocellular carcinoma cell lines. Despite the effective FAK degradation, these compounds still did not affect cell proliferation in any of the cell lines tested.

Recently, the Yu Rao group developed a FAK-targeting PROTACs with FAK inhibitors (PF562271 or VS6063) and CRBN ligand^244^ (Fig. 26). FC-11 (PF562271-based FAK PROTAC) showed picomolar FAK degradation in the tested cell lines (the DC_50_ in the tested cell lines were 40 pM in Ramos, 80 pM in PA1, 310 pM in TM3, 330 pM in MDA-MB-436 and 370 pM in LNCaP cell lines). However, like the other reported FAK PROTACs,^100,243^ FC-11 did not affect the cell proliferation in the tested cell lines to a greater extent than PF562271. Therefore, more work is required to study FAK-related biology.

### FLT-3

FMS-like tyrosine kinase 3 (FLT3), belongs to the type III RTK family, plays an important role in cell proliferation, differentiation and apoptosis.^245,246^ About 30% of newly diagnosed acute myeloid leukemia (AML) patients exhibit FLT3 mutations; these mainly include internal tandem duplication (ITD) mutations in 20–25% of AML cases and point mutations (e.g., D835) in the tyrosine kinase domain (TKD) in ~5–10% of cases.^247–249^ Both FLT3-ITD and TKD mutations result in continuous activation of FTL3 and loss of autoinhibitory function on FLT3, which ultimately promote the activation of the STAT, PI3K/Akt, and MAPK/ERK downstream signaling pathways.^250–252^ In recent years, much effort has been invested in the development of small-molecule FLT3 inhibitors. Quizartinib (AC220), gilteritinib, MLN-518, sunitinib and ponatinib are being studied in clinical trials.^253–257^ Although these FLT3 inhibitors exhibit potent activity against AML in clinical trials, acquired drug resistance and relapse still remain challenges for FLT3-targeted therapy.

**Fig. 27:**
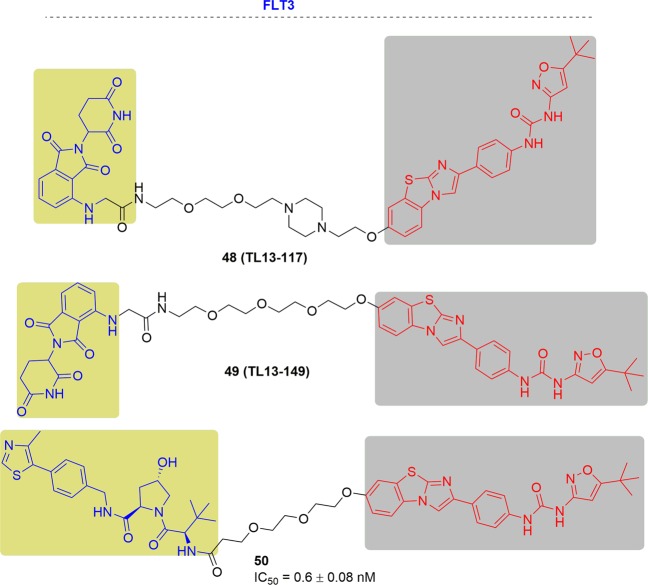
Representative PROTACs of FLT3.

In 2018, Nathanael S. Gray and his coworkers synthesized two FLT3-specific PROTACs, TL13-117 and TL13-149, based on the study of the multikinase degrader TL12-186. These specific FLT3-targeting PROTACs were synthesized by conjugating the clinical candidate quizartinib and the CRBN ligand pomalidomide with a PEG linker (Fig. 27).^169^ In MOLM-14 cells, TL13-117 and TL13-149 caused the most efficient FLT3 degradation at 10 to 100 nM respectively. However, quizartinib exhibited an approximately fivefold lower IC_50_ than TL13-117 and TL13-149 in both cells of MOLM-14 and MV4-11, indicating that TL13-117 and TL13-149 induced FLT3 degradation provide a little improvement to their antiproliferative effects.

In the same year, the Craig M. Crews group developed a FLT3 PROTAC by combining quizartinib and a VHL E3 ligand with an optimized linker (Fig. 27).^258^ This PROTAC displayed a low nanomolar degradation concentrations ofFLT3-ITD in MV4-11 and MOLM-14 cells, and the cell growth inhibition activity was >3.5-fold more potent than quizartinib with a subnanomolar IC_50_ (0.6 ± 0.08 nM) in contrast to the previously reported FLT3 PROTACs which failed to give an advantage.^169^ Additionally, the FLT3 PROTAC was capable of inducing FLT3 ITD degradation in MV4-11 xenograft tumors at the dosage of 30 mpk (the drug plasma levels were sustained at >5 nM during treatment).

### HDAC6

Histone deacetylases (HDACs) are a class of proteases whose main function is to modify the structure of chromosomes and regulate gene expression.^259^ HDAC6 belongs to the type II HDAC family, which has unique structural and biological properties.^260–262^ HDAC6 has two functional deacetylation domains and one zinc finger motif, which are required for HDAC6 to exert its biological activity. An increasing number of studies have shown that HDAC6 is closely related to the occurrence and development of tumors.^259,263,264^ HDAC6 inhibitors can inhibit cancer cell proliferation, promote apoptosis and have good effects in various malignant tumors, such as multiple myeloma (MM), non-Hodgkin lymphoma (NHL) and other malignant tumors.^262,265^ However, most HDAC6 inhibitors are poorly selective and act on a variety of HDAC isoforms, especially HDAC1 and HDAC3. Although they have obvious anti-differentiation and anti-proliferative effects, the side effects are also obvious, including myelosuppression, body mass loss, fatigue and arrhythmia, etc., which limits their serious utilization.^264,266,267^

**Fig. 28:**
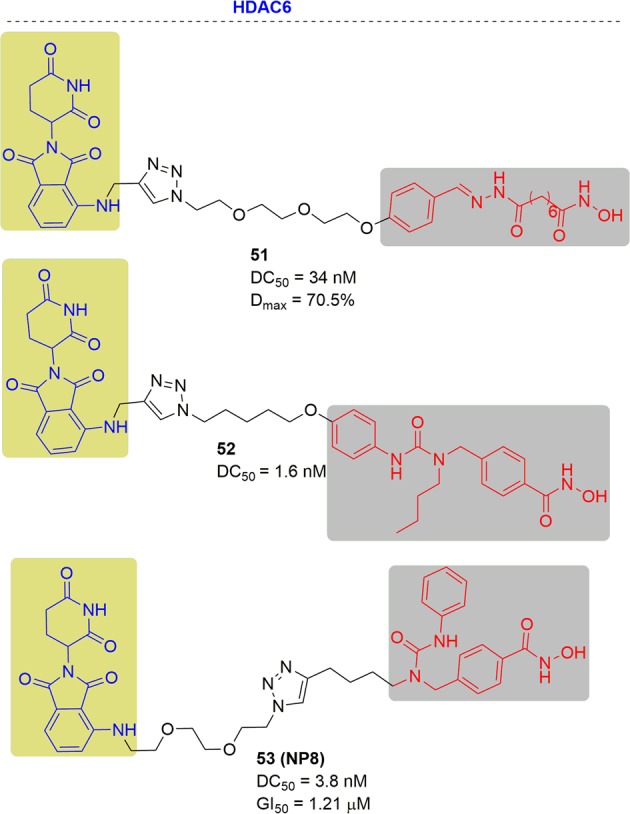
Representative PROTACs of HDAC6.

In 2018, Tang and coworkers designed and developed the first degrader for zinc-dependent HDACs by conjugating nonselective HDAC inhibitors with an E3 ubiquitin ligase^268^ (Fig. 28). In this work they found that the degradation from representative compound **51** occurred at 41 nM and reached the maximal effect ranging from 123 to 370 nM in MCF-7 cells. The DC_50_ and *D*_max_ were 34 nM and 70.5% respectively. hook effect was not observed at higher concentrations. They also found that the maximal effect of HDAC6 degradation was observed as low as 80 nM when they used compound **51** to treat the MM.1S cell line for 6 h.

In 2019, they reported a new generation of multifunctional HDAC6 degraders by tethering the selective HDAC6 inhibitor nexturastat A with a CRBN ligand that could be synergistic for the antiproliferation of MM^269^ (Fig. 28). In this work, they found that compound **52** reduced the HDAC6 level at a concentration as low as 3 nM and achieved maximal effects at approximately 30 nM. It showed a DC_50_ at approximately 1.6 nM in the MM.1S cell line, which was ~5- to 6-fold higher than compound **51**. At the same time, they found that compound **52** had a good selectivity for HDAC6, and showed less degradation of HDAC1, HDAC3, and HDAC4.

In 2019, Rao and coworkers reported developing potent PROTACs tools for selective degradation of HDAC6 protein (Fig. 28). They also chose nexturastat A (Nex A) as the HDAC6 binder, but they modified the PROTAC molecule to an alkyl chain instead of the benzene ring in compound **52.**^270^ In this study, representative compound **53** was the most potent degrader, which could significantly reduce the HDAC6 protein level at a concentration of 100 nM in HeLa cells. They also evaluated the degradation potential in various cell lines and found that compound **53** consistently induced significant degradation of HDAC6 in all cell lines but exhibited the best sensitivity in the MM cell line MM.1S. Moreover, they found that compound **53** had good selectivity for HDAC6, and had no degradation effects on HDAC1, HDAC2 or HDAC4, even at 10 μM. Compound **53** had a DC_50_ of 3.8 nM against HDAC6, and the GI_50_ was 1.21 μM in MM.1S cells. The degradation process was also well-illustrated by fluorescence-based visualization.

### MCL1

Myeloid cell leukemia 1 (MCL1) is a pro-survival protein overexpressed in a variety of different cancers, such as lymphoma, leukemia, breast cancer, and MM.^271^ MCL1 can combine with pro-apoptotic factors Bim, Bak, and Bax by PPI and silence their proapoptotic functions.^272^ Therefore, MCL1 has been regarded as a critical survival factor in human cancers. Considering that inhibition by traditional small molecules is dependent on occupation of the pocket at a certain concentration and for enough time, PPI are challenging to target due to their shallow binding regions. Since PROTACs can induce protein degradation without the need for high binding affinity, PROTACs hold great potential to overcome this problem.

**Fig. 29:**
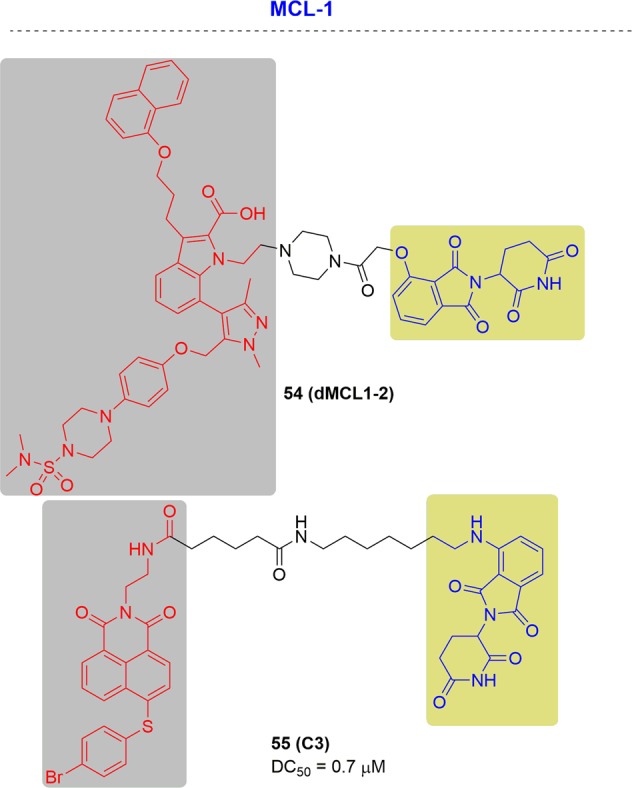
Representative PROTACs targeting MCL1.

In 2019, Derksen and coworkers designed dMCL1-2 and confirmed the ternary complex formation^273^ (Fig. 29). They proved that, compared with DMSO controls, dMCL1-2 could induce marked decreases in MCL1 levels at 100 nM in OPM2 cells by initiating MCL1 ubiquitination.

As mentioned above, MCL1 interacted with BCL2. Therefore, Zhang and coworkers also realized the degradation of MCL1 by PROTAC C1 with a DC_50_ value of 0.7 µM and achieved degradation of BCL2 at the same time^128^ (Fig. 29).

### MDM2

p53, a tumor suppressor, plays a pivotal role in many cellular processes regulation and the cancer development prevention.^274^ However, mutations or deletions of p53 occur in approximately 50% of human cancers resulting in inactivation of p53 tumor suppressor function.^275^ Murine double minute 2 (MDM2) is a negative endogenous cellular regulator of p53. As an E3 ligase, it could bind to and ubiquitinate p53, finally leading to efficient p53 degradation.^276^ Indeed, MDM2 is overexpressed in some human p53 wild-type cancers. To restore the tumor suppressor function of p53, disruption of MDM2-p53 interactions has become a promising therapeutic strategy for p53 wild-type human cancers. However, p53 inhibition leads to the overexpression and accumulation of MDM2, which may lead to toxicity issues. In addition, despite the significant progress in the development of MDM2 inhibitors, drug resistance has become a significant limitation. Thus, the PROTAC strategy has become a highly desirable method for the modulation of MDM2 levels.

**Fig. 30:**
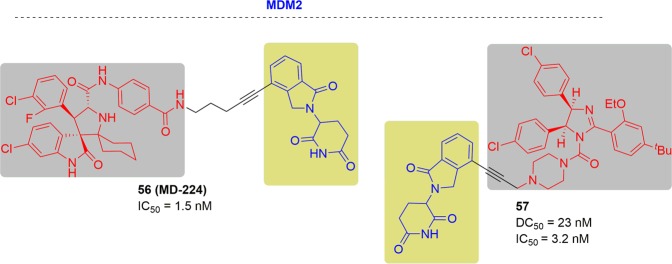
Chemical structures of the reported MDM2 inhibitor and PROTACs.

In 2018, the Shaomeng Wang group published the first potent MDM2 degrader, MD-224, by tethering the spirooxindole MDM2 inhibitor MI-1061 to the CRBN ligand lenalidomide^61^ (Fig. 30). MD-224 effectively induced MDM2 degradation at subnanomolar concentrations in human leukemia cells. It achieved an IC_50_ value of 1.5 nM for inhibiting the growth of RS4;11 cells and other leukemia cell lines. In addition, MD-224 also exhibited complete and durable tumor regression in vivo, which outperformed the inhibitor MI-1061.

In 2019, the Weiping Tang group reported the second MDM2 degrader, degrader 32, through connection of MDM2 ligand (nutlin) and CRBN E3 ligase ligand (lenalidomide) (Fig. 30).^277^ Degrader 57 induced efficient degradation of MDM2 with a DC_50_ value of 23 nM in RS4;11 leukemia cells. It also inhibited leukemia cells proliferation with IC_50_ of 3.2 nM, which was nearly 1000-fold more potent than MDM2 inhibitor.

### p38α and p38δ

The p38 MAPK kinases are activated by various cellular stresses and inflammatory cytokines.^278,279^ They consist of four members (p38α, p38β, p38γ, and p38δ) but rare isoform-selective chemical probes have been reported.^47^ p38 MAPKs are activated via dual phosphorylation of their Thr–Gly–Tyr motif in their activation loop and subsequent characteristic global conformational changes. Among the isoforms, p38α is the best studied isoform and thus many inhibitors were developed for it, but they showed limited efficacy and safety. In contrast, p38δ has been under studied for cancer and diabetes and its functional inhibition seems to be intractable.

**Fig. 31:**
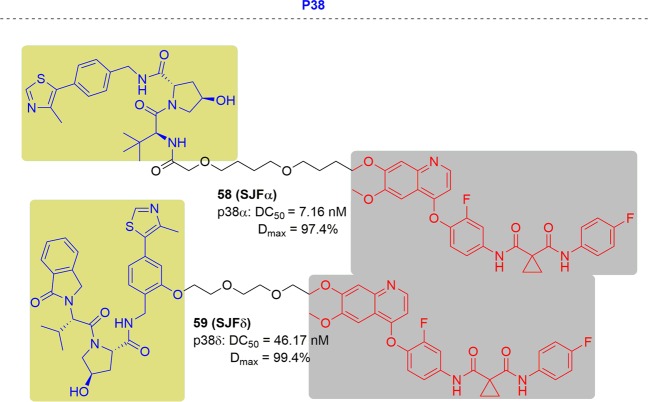
Representative PROTACs of p38α and p38δ.

The Crews group developed p38α- and p38δ-selective PROTACs based on foretinib and different E3 ligase (VHL) ligands^47^ (Fig. 31). SJFα degraded p38α with a DC_50_ of 7.16 nM and a *D*_max_ of 97.4%, while it was less effective against p38β, p38γ and p38δ. SJFδ degraded p38δ with a DC_50_ of 46.17 nM and a *D*_max_ of 99.4%, while it did not degrade p38α, p38β or p38γ. They then used an in vitro ternary complex pull-down assay to demonstrate the selectivity. It was found that SJFα only facilitated the VHL:PROTAC:p38α ternary complex, whereas no such ternary species was detected in the presence of SJFδ. However, both SJFα and SJFδ could engage in VHL:PROTAC:p38δ ternary complexes with similar efficiency. Thus, they used surface plasmon resonance (SPR) to study the assembly kinetics of the binary and ternary complexes. The SJFδ complex showed an increased half-life (*t*_1/2_ = 38 s) compared with the SJFα complex (*t*_1/2_ = 8 s), which indicated that p38δ:SJFδ:VHL is the more favorable ternary complex compared with p38α:SJFδ:VHL, which corresponds to the degradation outcomes. Molecular dynamics (MD) simulations on the p38δ:SJFδ:VHL and p38δ:SJFα:VHL complexes revealed how linker length and orientation for recruiting VHL on different PROTACs can result in different ternary interfaces to achieve selective degradation.

### PARP1

Poly (ADP-ribose) polymerases (PARPs) belongs to DNA-dependent nuclear enzymes, which plays the role of transfer negatively charged ADP-ribose moieties from cellular NAD+ to different protein substrates.^280^ PARP1 is the most abundant nuclear enzyme of the PARP family. It possesses the functions of repairing DNA damage due to replication, exposure to exogenous toxins, ionizing radiation, ultraviolet radiation, environmental factors, chemotherapy, cellular metabolites, radiotherapy, etc. and plays a role in stopping cell death.^281^ Due to the pivotal role of PARP1 in the DNA damage response, it is regarded as a potent cancer therapeutic target. Currently, a number of PARP1 inhibitors, such as olaparib, niraparib and iniparib, are in different stages of clinical trials.^282^ However, cytotoxicity and drug resistance are the biggest obstacles for their use in patients. Thus, other therapeutic method with novel action mechanisms are remain highly needed.

**Fig. 32:**
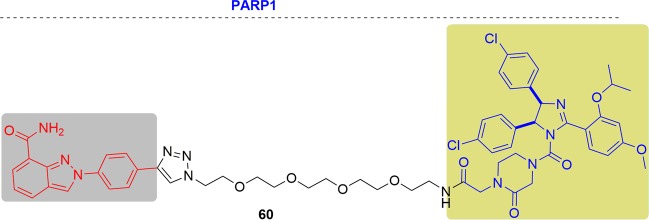
Representative PROTAC of PARP1.

In 2018, the Yu Rao group published the first PARP1-targeting PROTAC (compound **60**) by connecting the PARP1 inhibitor niraparib and the MDM2 ligand nutlin-3^283^ (Fig. 32). After a broad degradation screening in several triple negative breast cancer (TNBC) cell lines, it was found that compound **60** could selectively induce significant PARP1 degradation and cell apoptosis in MDA-MB-231 cells. Furthermore, compound 3 is fivefold more potent than PARP1 inhibitors (niraparib, olaparib and veliparib) in terms of the antiproliferative activity and showed no cytotoxicity in normal cells.

### PI3K

Phosphoinositide 3-kinases (P13Ks) are intracellular phosphatidylinositol kinases which were the members of PI3K/Akt/mTOR signaling pathway, which are involved in the regulation of cell proliferation, apoptosis and differentiation.^284–286^ Recent studies have found that overexpression of the P13K-dependent signaling pathway is a major feature of tumorigenesis. According to different structures and functions, PI3Ks are normally classified into three classes, among which class I PI3Ks are considered to be most closely related to tumor development and the most commonly studied enzyme.^287–290^ Class I PI3Ks can be divided into IA (PI3Kα, PI3Kβ, and PI3Kδ) and IB (PI3Kγ). Although many PI3K inhibitors have already been developed, their drug-like properties are seriously limited due to poor selectivity and side effects.^291–293^ Moreover, the high mutation rate of the PIK3CA gene, which encodes PI3Kα in solid tumors, makes the development of PI3Kα inhibitors more difficult.^294,295^ Therefore, the development of novel protein degradation agents targeting the PI3K protein has become an excellent strategy.

**Fig. 33:**
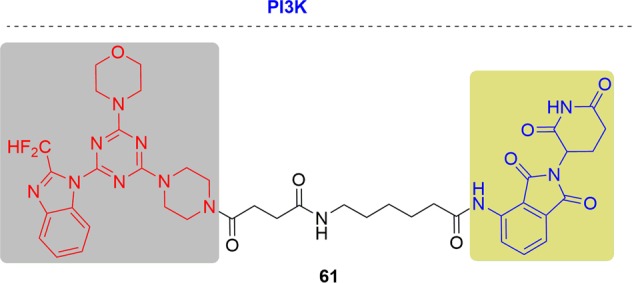
Representative PROTAC of PI3K.

Jiang and coworkers designed and synthesized a series of potential PROTACs based on CRBN and **ZSTK474** for the degradation of PI3K^296^ (Fig. 33). Representative compound **61** induced remarkable PI3K degradation at 10 μM, and the phosphorylation of Akt, S6K, and GSK-3β in the PI3K/Akt/mTOR signaling pathway could also be downregulated in HepG2 cells. However, the enzymatic activity of representative compound **61** against PI3Kα proved to be worse than the control ZSTK474.

### Pirin

Pirin is an iron-binding member of the cupin super family of proteins and has been reported to be a transcription factor regulator.^297^ It interacts with BCL3, the proto-oncoprotein, linking with the NFκB pathway via a pirin/p65/DNA complex. It was hypothesized that the role of human pirin to be a redox-sensing transcription factor regulator under the control of the transcript factor NRF2 through changes in cellular oxidative stress. The knockdown of pirin via siRNA could suppress cancer cell migration and proliferation.^298,299^

**Fig. 34:**
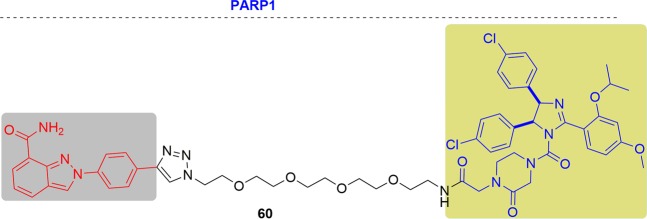
Representative PROTAC of pirin.

Jones and coworkers reported a high affinity pirin chemical probe CCT251236 via a phenotypic screen to develop pirin-targeting PROTACs^300^ (Fig. 34). Their first generation PROTACs possessed good affinities for pirin and the CRBN-DDB1 complex, but there was no observable degradation of pirin or effects on cancer cells. They hypothesized that the physicochemical properties were the main cause of the failure of the first generation PROTACs. They reduced the tPSA and HBD count while maintaining an acceptable Log D of 7.4 to balance the permeability and solubility in the second generation PROTACs that gave encouraging results. However, the CRBN-targeting thalidomide ligand in the PROTACs rapidly decomposed at 37 °C in pH 7.4 phosphate buffer. Therefore, they aimed to optimize the permeability further in the third generation to result in higher free intracellular concentrations more quickly. Under the treatment of the third generation pirin-targeting PROTAC CCT367766, nearly complete pirin degradation could be observed with just 50 nM treatment and only 2 h of exposure. The whole proteome mass spectrometry showed good selectivity of PROTAC CCT367766.

### PRC2 (EED-targeted)

Polycomb repressive complex 2 (PRC2) includes three core subunits: embryonic ectoderm development (EED), enhancer of zeste homolog 1 (EZH1) or EZH2, and suppressor of zeste homolog 12 (SUZ12).^301^ PRC2 has histone methyltransferase (HMT) activity that installs and maintains mono- to tri-methylation at lysine 27 of histone 3 (H3K27). The intricate network of protein–protein interactions between EED, EZH2, and SUZ12 are necessary for PRC2 catalytic activity.^302–304^ It has been reported that PRC2 behaves as both an oncogene and a suppressor of tumorigenesis in a variety of cancer types. EZH2, EED, and SUZ12 are commonly upregulated and are also susceptible to mutations in certain cancers such as breast, colorectal, and prostate cancer.^302,305^ Thus, targeting PRC2 for cancer treatment has become an effective strategy. Currently, effective inhibition of PRC2 catalytic activity has been achieved by targeting both EED and EZH2. Despite several inhibitors of EZH2 (e.g., UNC1999, GSK126, EPZ-6438, CPI-1205, and DS-3201b) and EED (e.g., EED226, A-395, and MAK683) in clinical development, drug resistance has been observed and is a limitation for this class of molecules. Therefore, new approaches are needed to overcome the observed resistance.

**Fig. 35:**
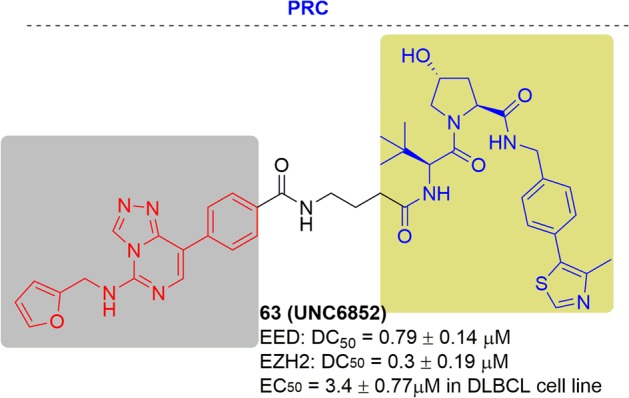
Representative PROTAC of PRC2.

The Lindsey I. James group first reported a chemical PRC2 degrader, UNC6852, which contained an EED226-derived ligand and a VHL ligand (Fig. 35).^306^ UNC6852 potently degraded EED and EZH2 with DC_50_ values of 0.79 ± 0.14 μM and 0.3 ± 0.19 μM, respectively, while SUZ12 showed less degradation. In addition, UNC6852 blocked the histone methyltransferase activity of EZH2 and inhibited the proliferation of DB cells (a DLBCL cell line harboring the EZH2 Y641N mutant) with an EC_50_ of 3.4 ± 0.77 μΜ after 9 days of treatment, which was similar to the inhibitors EED226 and UNC1999

### RIPK2

The serine-threonine kinase RIPK2 is an important innate immune mediator of NOD1 and NOD2 signaling.^307^ NOD1 and NOD2 are cytosolic receptors for bacterial peptidoglycan derivatives such as muramyl dipeptide (MDP), which are associated with activated RIPK2 and recruit kinases such as TAK1, IKKα, IKKβ, and IKKγ for NF-κB and MAPK activation. This results in the expression of a variety of inflammatory proteins and anti-bacterial proteins, activation of autophagy and antigen presentation. The NOD–RIP2 signaling pathway is particularly relevant to intestinal inflammation and mucosal immunity in the respiratory system, which is regulated by ubiquitination.

**Fig. 36:**
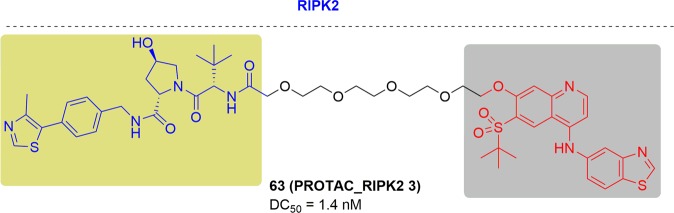
Representative PROTAC of RIPK2.

The Crews group developed the RIPK2-targeting PROTAC molecule PROTAC_RIPK2 (**63**), which gave a *D*_max_ of >95% at concentrations of more than 10 nM, and a DC_50_ of 1.4 nM^205^ (Fig. 36). They then addressed one of the facets of PROTAC action: substoichiometric catalysis, whereby one PROTAC molecule is able to induce the ubiquitination and degradation of multiple target protein molecules. To determine the catalytic nature of PROTAC_RIPK2, they determined the absolute amount of RIPK2 by liquid scintillation analysis in reactions containing 0.50, 1.0, and 2.0 mol of PROTAC, which resulted in 1.7, 3.4, and 4.0 pmol of modified RIPK2, respectively, corresponding to stoichiometries of 3.3, 3.4, and 2.0. These data provide evidence for the catalytic manner of degradation by PROTACs. However, they thought that the catalytic ability was underestimated for the cellular environment and polyubiquitination. A cellular quantitative expression proteomic study also revealed high specificity for target degradation.

### Rpn13

Rpn13 is associated with the 19S regulatory component of the proteasome and captures ubiquitinated proteins as a substrate for degradation via the 20S proteasome.^308^ The ubiquitin moieties are then removed from the captured substrate by deubiquitinating enzyme UCH37 at the 19S proteasome, which is then unfolded by the AAA-ATPases for further 20S proteasome-mediated degradation. Inhibition of proteasome is an effective strategy for treating multiple myeloma (MM) but targeting different components of the ubiquitin–proteasome system remains elusive. Rpn13 expression is higher in MM cells and plays important roles in MM cell growth and survival. Both RNA interference and inhibitors confirmed that Rpn13 was a drug target for MM.^309^

**Fig. 37:**
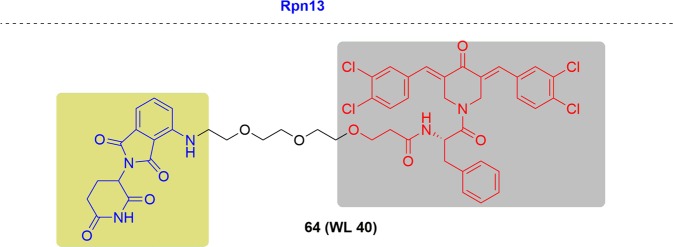
Representative PROTAC of Rpn13.

Chauhan and coworkers designed a degrader, WL-40, by linking the Rpn13 covalent inhibitor RA190 with a CRBN ligand^309^ (Fig. 37). The covalent binding of RA190 to Rpn13 could block the recognition of polyubiquitinylated proteins for subsequent degradation by the proteasome. In WL40-treated cells, the levels of Rpn13 were maximally (95%) reduced after 16 h. Importantly, in contrast to bortezomib, which could selectively inhibit 20S proteasomal activities and therefore lead to the aggregation of lower molecular weight polyubiquitinated proteins, WL40 only blocked the 19S proteasome and prevented the deubiquitylation of substrates, thereby resulting in higher molecular weight polyubiquitinated proteins. Furthermore, WL-40 triggered potent anti-MM activity in the presence of a cytoprotective tumor BM microenvironment, overcame bortezomib resistance and was active in the context of mutated p53. WL40 induced the ER stress response/UPR and p53/p21 apoptotic signaling faster than RA190. In addition, the in vivo study showed that WL40 significantly inhibited tumor growth with half the equimolar dose of RA190.

### SGK3

Serum/glucocorticoid-inducible protein kinase (SGK) is a key downstream signaling molecule of PI3K, which plays an important role in the regulation of cell proliferation, survival, invasion and metastasis.^310^ SGK-3, an isoform of the SGK family, plays an important role in cell proliferation and survival, especially in breast cancer, liver cancer, colorectal cancer, and prostate cancer.^311,312^ Recent studies have found that SGK-3 is overexpressed in breast cancer cells and that its inhibitor could significantly inhibit the proliferation of breast cancer cells. However, the currently known SGK-3 inhibitors have poor IC_50_ values and selectivity.^313^ Thus, it seems that optimization and characterization of an SGK3-specific PROTAC is particularly attractive.

**Fig. 38:**
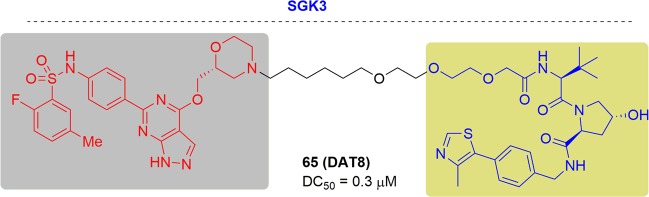
Representative PROTAC of SGK-3.

Dario R. Alessi and coworkers first designed a PROTAC conjugate of the 308-R SGK inhibitor with the VH032 VHL binding ligand, targeting SGK3 for degradation^314^ (Fig. 38). Compound **65** induced 50% degradation of the endogenous SGK3 at 0.3 μM within 2 h, and maximal 80% degradation was observed within 8 h in HEK293 cells. In contrast to the inhibitor, the degrader had good selectivity, which did not degrade the closely related SGK1 and SGK2 isoforms. The degrader can also suppress proliferation of ZR-75-1 and CAMA-1 cancer cell lines compared with a PI3K inhibitor (GDC0941).

### Smad3

Smad3 is an important transporter in the transforming growth factor β (TGF-β) signaling pathway, which is responsible for the direct transfer of the TGF-β signal from the extracellular space to the nucleus and regulation of the expression of target genes.^315,316^ It is known that the expression level and functional status of Smad3 affects the signal transduction process, involving cell growth, proliferation, development, differentiation, migration and apoptosis.^316–318^ It has been confirmed that the overexpression of Smad3 is related to liver fibrosis and renal fibrosis, especially in a variety of kidney diseases, such as obstructive nephropathy, diabetic nephropathy, and hypertensive nephropathy. Therefore, the strategy to construct a new proteolysis-targeting chimeric molecule (PROTAC) that may prevent kidney fibrosis by targeting ubiquitination and degradation of basic intracytoplasmic Smad3 is of great significance.

**Fig. 39:**
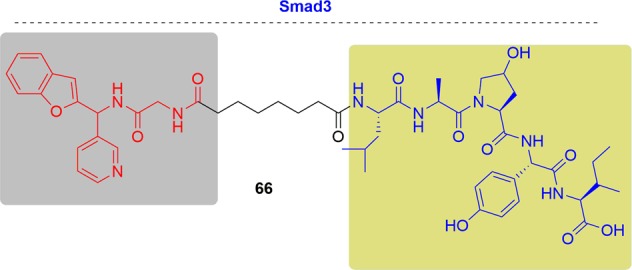
Representative PROTAC of Smad3.

Wang and coworkers screened small-molecule ligands that bind to Smad3 and then used the molecule to synthesize a target compound^319^ (Fig. 39). They observed slight degradation after treatment with 1 μg of **66** in ACHN cell lysates.

### STAT3

STAT3 belongs to the STAT family, which contains seven members including STAT1, STAT2, STAT3, STAT4, STAT5A, STAT5B, and STAT6. They share a conserved Src-homology 2 (SH2) domain, which accounts for homodimerization through phosphorylated Tyr 705 and subsequent transactivation. Therefore, inhibitors targeting the SH2 domain to disrupt the PPIs for homodimerization have been limited by the homologous selectivity. On the other hand, only partial transcriptional activity of STAT3 could be suppressed because monomeric STAT3 still remains active. Although embryonic lethality is observed in STAT3 null mice, primary mouse fibroblasts grow at a near speed in STAT3 null mice as wild-type fibroblasts, which indicates that STAT3 is dispensable in normal cells.^320^ However, as a transcriptional factor, STAT3 plays a critical role in oncogenesis by regulating genes related to cell survival, proliferation, invasion, metastasis. STAT3 has been proposed as a particularly attractive target for potential cancer therapy.

**Fig. 40:**
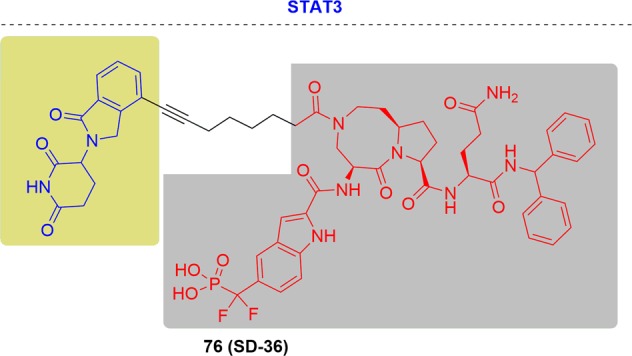
Representative PROTAC of STAT3.

Given that developing effective and selective inhibitors of STAT3 remains challenging, very recently, the Wang group developed potent and specific PROTAC degraders targeting STAT3 that showed great in vivo therapeutic potential for AML and anaplastic large-cell lymphoma (ALCL)^321^ (Fig. 40). They first performed optimization based on their previous STAT3 SH2 domain inhibitor CJ-887 and obtained a ligand named SI-109, with high affinity for STAT3 and good cell permeability. The cocrystal structure of STAT3 with SI-109 was solved and guided the design of PROTACs by tethering SI-109 and an analog of lenalidomide. The degrader SD-36 demonstrated high activity in AML and ALCL cells. It degraded >90% STAT3 in AML cells within 4 h and >50% STAT3 in ALCL cells. A series of rescue experiments confirmed that SD-36 is a bona fide PROTAC and not a molecular glue. More importantly, SD-36 showed excellent selectivity, as other members of the STAT family could not be degraded or bound. Some STAT3 mutations, such as D661Y, K658R, and Y705F, were also effectively degraded by SD-36. SD-36 depleted both monomeric and dimeric STAT3 in AML cells at the concentration of 1 μM after treatment for 5 h; thus, the transcriptional activity of STAT3 was potently and specifically inhibited. For example, its downstream genes, such as BCL3, HCK, HGF, JAK3, PIM1, SOCS3, and VEGFA, were all downregulated. SD-36 induced apoptosis by caspase-3/7 activation and PARP cleavage; meanwhile, the level of STAT3 in mitochondria was also reduced. SD-36 effectively induced the degradation of STAT3 xenograft tumors and achieved complete and long-lasting tumor regression in mice. After the analysis of other mouse tissues, such as the liver, spleen, heart, and kidney, it was found that SD-36 caused a profound depletion of STAT3 in these tissues, but its safety appeared to be good.

### TBK1

TANK-binding kinase 1 (TBK1) is a serine/threonine kinase and a noncanonical member of the IKK family with many cellular functions in innate immunity, tumorigenesis and development.^322^ More interestingly, some RNAi experiments have indicated the K-Ras synthetic lethality with TBK1.^323^ However, subsequent reports have challenged this hypothesis.^324^

**Fig. 41:**
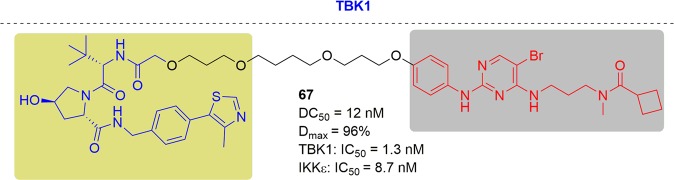
Representative PROTAC of TBK1.

Instead of RNAi, the Crews group designed TBK-targeting PROTACs that could be used as tools for investigating the relationship between TBK1 and K-Ras mutants^322^ (Fig. 41). They selected a crystal structure of TBK1 bound to an inhibitor as the starting point. After a structure-based modification with the available structure-activity relationship (SAR), the inhibitor was used as the protein targeting moiety of the VHL PROTACs. A systematic survey of linker length indicated that PROTACs with linkers of less than 12 atoms demonstrated no appreciable degradation activity due to the steric conflicts in the ternary complex. Representative PROTAC **67** with a linker of 15 atoms showed high cellular degradation potency (DC_50_ = 12 nM) and maximum degradation (*D*_max_ = 96%). They further modified the protein-targeting moiety and VHL ligand to study more SAR. The ability of PROTAC **67** to degrade noncanonical IkB kinase IKKε, a close homologue of TBK1, was tested. Although the inhibitor exhibited poor selectivity for TBK1 over IKKε (IC_50_ values of 1.3 nM vs 8.7 nM), PROTAC **67** had no effect on the level of IKKε. They hypothesized that the selectivity resulted from the differential presentations of their surface lysines to VHL and its reactive E2 ubiquitin thioester component, therefore resulting in different efficiency of the transfer of ubiquitin. In addition, the difference in the formation and stability of the ternary complex might contribute to the selectivity. Finally, the cell proliferation effect of PROTAC **67** in both K-Ras mutant cell lines (H23, A549, and H1792) and K-Ras wild-type cell lines (H2110 and HCC827) was evaluated. The results showed that there was no significant difference in these cells, which indicated that TBK1 was not synthetically lethal in the K-Ras mutant.

### TRIM 24

The addressable pocket of a protein is often not functionally relevant in disease. This is true for the multidomain, bromodomain-containing transcriptional regulator TRIM24. TRIM24 has been posited as a dependency in numerous cancers, yet potent and selective ligands for the TRIM24 bromodomain do not exert effective anti-proliferative responses.^325^

**Fig. 42:**
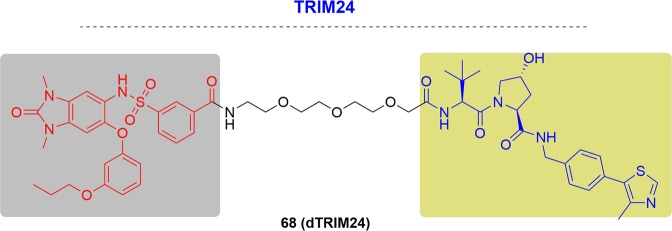
Representative PROTAC targeting TRIM 24.

In 2018, the Bradner group developed a PROTAC, dTRIM24, to degrade TRIM24 by recruiting the VHL E3 ubiquitin ligase^326^ (Fig. 42). The degrader elicited potent and selective degradation of TRIM24 with a maximum degradation at 5 µM. Using dTRIM24 to probe TRIM24 function, they characterized the dynamic genome-wide consequences of TRIM24 loss on chromatin localization and gene control. Furthermore, they identified TRIM24 as a novel dependency in acute leukemia. A pairwise study of TRIM24 degradation versus bromodomain inhibition revealed an enhanced antiproliferative response from degradation. dTRIM24 offered a chemical probe of an emerging cancer dependency and established a path forward for numerous selective yet ineffectual ligands for proteins of therapeutic interest.

## PROTACs targeting virus-related targets

### NS3

The hepatitis C virus (HCV) NS3 protein plays multiple essential roles in viral infection, such as those of a serine-type protease (N-terminus of NS3 with cofactor NS4A) and a helicase (C-terminal of NS3). At present, telaprevir (VX-950) was approved for the treatment of HCV as a reversible covalent NS3/4A protease inhibitor. In contrast, HCV patients have formed drug resistance after receiving telaprevir treatment due to the low barrier of this type inhibitor. Consequently, telaprevir was taken off the market.

**Fig. 43:**
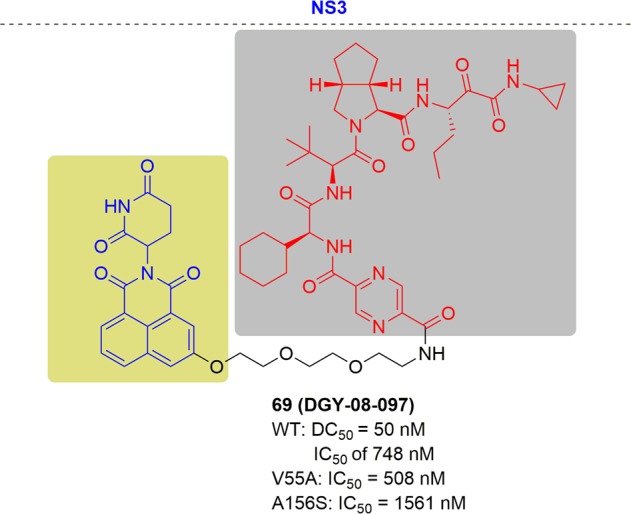
Representative PROTAC targeting NS3.

Yang and coworkers first developed a series of novel PROTACs for NS3 degradation^327^ (Fig. 43). The representative degrader DGY-08-097 could efficiently degrade NS3 (50% degradation efficiency at 50 nM after 4 h in cells that induce full-length HCV NS3 protein expression). DGY-08-097 had an IC_50_ of 748 nM, which is worse than that of telaprevir (IC_50_, 132 nM) against wild-type NS3 (Huh7.5 cells). In the mutant NS3 system, DGY-08-097 could degrade both V55A and A156S mutant NS3, with IC_50_ values of 508 nM (V55A) and 1561 nM (A156S). Compared with telaprevir (IC_50_: V55A 288 nM; A156S 949 nM), DGY-08-097 is less sensitive to mutations. These results demonstrate that targeted protein degradation is a novel antiviral strategy.

## PROTACs for treating immune disorders

### IRAK4

Interleukin-1 receptor-associated kinase 4 (IRAK4) is a key molecule that participates in innate immune processes. It belongs to the IRAK family, which contains four serine/threonine kinases IRAK4, IRAK1, IRAK2, and IRAK-M.^328^ It has been demonstrated that the loss of function or deficiency of IRAK4 would increase susceptibility to pathogens, while over activation of IRAK4 is linked with some autoimmune diseases.^329^ Although some inhibitors have blocked kinase activity in clinical trials, they still cannot achieve the desired effect, since several reports have indicated that the nonkinase functions or scaffolding functions played more important roles than the kinase function in certain cell types.

**Fig. 44:**
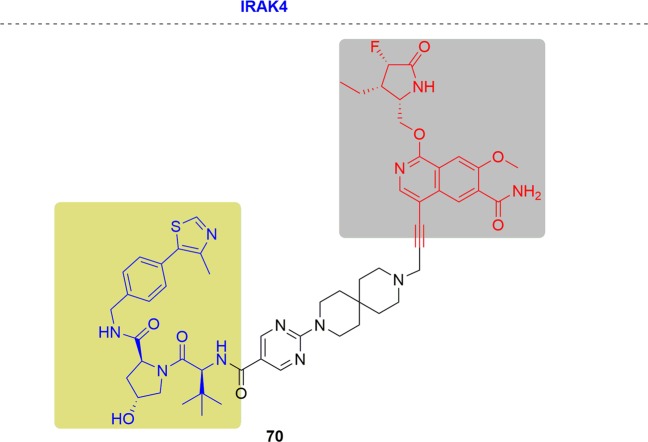
Representative PROTAC of IRAK4.

Hence, researchers from GlaxoSmithKline designed IRAK4-targeting PROTACs based on PF-06650833 to remove all protein functions, including the kinase-dependent functions and scaffold functions, which was likely to achieve wider pharmacological effects than inhibitors^329^ (Fig. 44). E3 ligase ligands of VHL, CRBN, and IAP were all tested in their work, while only VHL PROTACs were found to degrade IRAK4. A hydrophobic all-carbon chain was more suitable as a linker than the hydrophilic PEG due to its permeability. After a small modification with the more rigid, polar spirocyclic pyrimidine, compound **70** was obtained with better solubility, a more potent DC_50_ value (151 nM in peripheral blood mononuclear cells and 36 nM in dermal fibroblasts), and a lower in vitro clearance in liver microsomes. However, the PROTAC (compound **70**) did not possess a different pharmacological profile than the inhibitor (PF-06650833) in the present work, which suggested that more studies are required to understand the biology of IRAK4.

### PCAF/GCN5

P300/CBP-associated factor (PCAF) and general control nonderepressible 5 (GCN5) are considered epigenetic proteins, because they both have acetyltransferase functions and a bromodomain. PCAF and GCN5 play multiple important roles in several cellular pathways for DNA damage repair, metabolic regulation, and cell proliferation and differentiation. Currently, GSK4027 is a bromodomain inhibitor of PCAF and GCN5. However, lipopolysaccharide (LPS) does not induce significant dose-dependent changes in several different inflammatory cytokines after treatment with GSK4027 in macrophages. However, the change phenomena can be observed in the PCAF^−/−^ model.

**Fig. 45:**
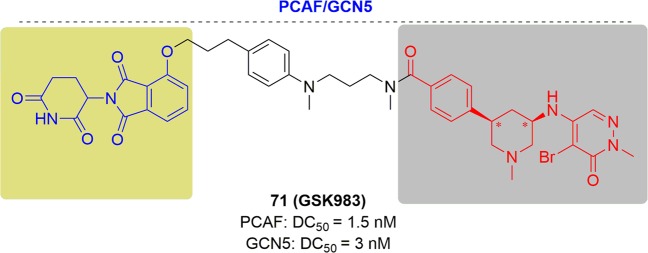
Representative PROTAC of PCAF/GCN5.

Tough and coworkers first developed a series of novel PROTACs for PCAF and GCN degradation^330^ (Fig. 45). The mixture of diastereomers degrader GSK983 could efficiently degrade both PCAF (50% degradation efficiency at 1.5 nM in THP1 cells) and GCN5 (50% degradation efficiency at 3 nM). The *cis*-(*R,R*)-enantiomer GSK699 can also efficiently degrade PCAF/GCN5. The mediated degradation of PCAF/GCN5 significantly decreased the ability of dendritic cells (DCs) and macrophages to respond to LPS, further reducing the production of many inflammatory cytokines. These results proved that the PCAF/GCN-targeting PROTAC degraders displayed distinct advantages over PCAF/GCN bromodomain inhibitors for a novel anti-inflammatory therapeutic strategy.

### Sirt2

Sirtuins and its cofactor NAD+ constitute the 18 different class III lysine deacetylases (KDCA). Sirtuins not only have the function of deacetylation, but they also remove the other acyl groups of side chain amino groups of acylated lysine, including palmitoyl, myristoyl, succinyl, glutaryl, and crotonyl groups. AN imbalance in Sirtuin2 (Sirt2) is related to the occurrence of various diseases, such as neurodegenerative diseases, cancer, type II diabetes, and bacterial infections.

**Fig. 46:**
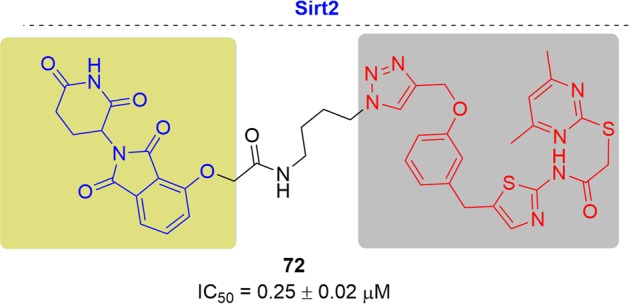
Representative PROTAC of Sirt2.

Jung and coworkers developed a novel selective inhibitor of Sirt2^331^ and utilized the inhibitor designed for Sirt2 as a targeting PROTAC^332^ (Fig. 46). The degrader **72** could efficiently inhibit the activity of Sirt2 (IC_50_ value of 0.25 ± 0.02 μM) but not the isotypes Sirt1 and Sirt3. The degradation of Sirt2 treated with degrader **72** showed a dose-dependent manner at concentrations of 0.05–5 μM. Compared with the reported inhibitor, **72** induced more obvious tubulin acetylation with enhanced process elongation. These results proved that the Sirt2-targeting PROTAC displayed distinct advantages over Sirt2 inhibitors; moreover, this is the first work for the epigenetic eraser protein PROTAC.

## PROTACs for treating neurodegenerative disease

Tau plays an essential role in neuronal cells to stabilize microtubules (MTs), provide tracks in the transport of cargo proteins and maintain cell shape. Dysregulation of Tau is an important characteristic in a variety of neurodegenerative diseases, such as Alzheimer’s disease (AD) and frontotemporal dementia (FTD) and also mediated the toxicity of amyloid-β (Aβ). Tau is a nonenzymatic protein and there are currently no good small molecules to address with its imbalance.

Although the Li and Jiang groups have developed PROTACs that target polypeptide types that degrade the Tau protein, the druggability of peptide-based degraders remained to be considered.^333,334^

**Fig. 47:**
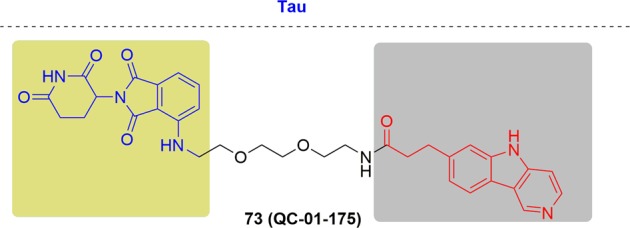
Representative PROTAC of Tau.

Haggarty and coworkers developed a series of novel PROTACs for Tau degradation^335^ (Fig. 47). The representative degrader QC-01-175 could efficiently degrade both wild type and variant Tau between 0.01 µM and 10 µM in neurons. Surprisingly, QC-01-175 can preferentially degrade Tau in FTD neurons compared to healthy cells. These results may provide a new strategy for the degradation of Tau with PROTACs to treat neurodegenerative disease.

## Other PROTACs

### FKBP12

FK506 binding protein 12 (FKBP12) binds to the Ca^2+^-release channel (ryanodine receptors, RyRs), which makes the calcium channel in a stable closed state. After FKBP12 dissociates from the RyRs, the RyRs open and release calcium ions, thereby regulating the organism through the Ca^2+^ signal pathway. One of the important functions of FKBP12 is to participate in cardiac development, which plays essential roles in regulating the phenotypic differentiation of cardiac cells, the formation of cardiac structure and the initiation of heart beats. Deletion of FKBP12 in the embryonic heart causes severe developmental ventricular defects, leading to embryonic death.

**Fig. 48:**
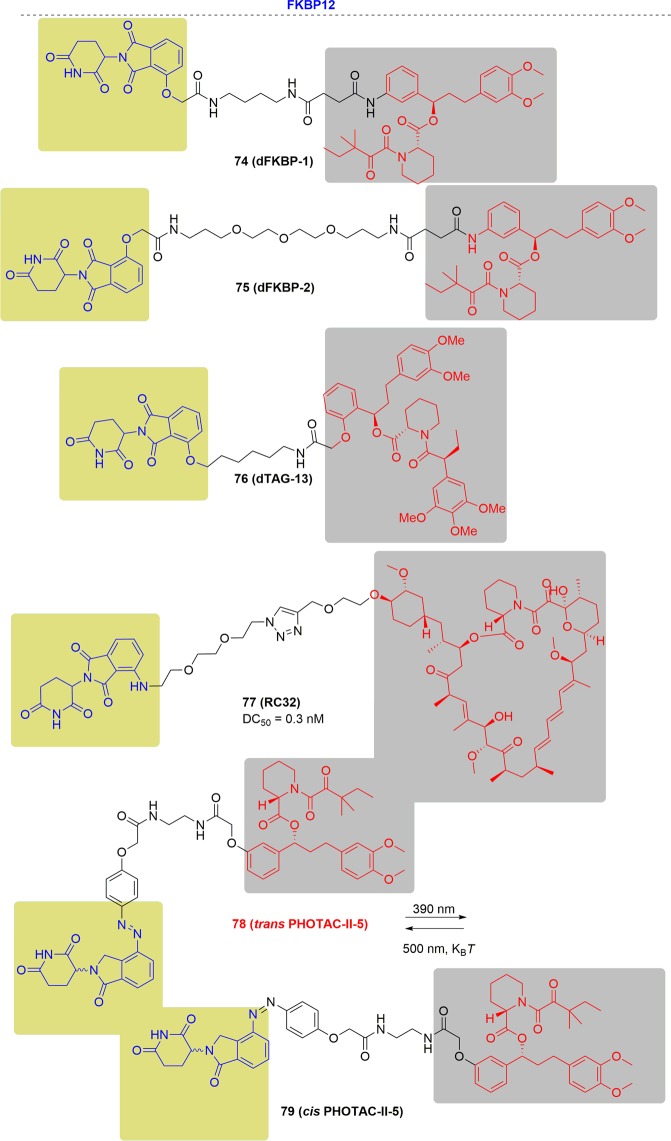
Representative PROTACs of FKBP12.

In 2015, the Bradner group designed and synthesized a FKBP 12-targeted PROTAC using CRBN ligands and a FKBP12^wild-type^ inhibitor^336^ (Fig. 48). They designed and synthesized two PROTACs, dFKBP-1, and dFKBP-2, which showed significant degradation of FKBP12 between the concentrations of 0.01 µM and 10 µM in MV4-11 cells. However, they did not further study the biological functions of the PROTACs beyond the degradation of FKBP12.

Subsequently, in 2018, Bradner and his colleagues continued to develop a series of PROTAC molecules targeting FKBP12 with the CRBN ligand thalidomide and the FKBP12^F36V^ selective inhibitor AP1867, of which dTAG-13 showed high selectivity and efficiency for FKBP12^F36V^ degradation^337^ (Fig. 48). Furthermore, the constructed exogenous FKBP12^F36V^ fusion proteins FKBP12^F36V^-BRD4, FKBP12^F36V^-KRAS^G12V^, FKBP12^F36V^-EZH2, HDAC1-FKBP12^F36V^, MYC-FKBP12^F36V^, and PLK1-FKBP12^F36V^ were also well degraded by dTAG-13. In addition, dTAG-13 successfully degraded FKBP12 in xenograft mice stably expressing luciferase-FKBP12 MV4-11 cells in vivo. The work of the dTAG system not only revealed the physiological roles of BRD4 and KRAS^G12V^ in detail but also provided a novel strategy for target validation during new drug development.

Rao and coworkers developed a novel FKBP12 targeting PROTAC, RC32, with the ligands pomalidomide and rapamycin^167^ (Fig. 48). Degrader RC32 showed efficient FKBP12 degradation with a DC_50_ of ~0.3 nM in Jurkat cells in vitro. Importantly, this work first demonstrated that RC32 could achieve the systemic knockdown of FKBP12 in animals (mouse, rat, Bama pig and rhesus monkey) in vivo, and the protein FKBP12 could also be gradually recovered after withdrawing drug administration. In addition, RC32 could still maintain highly efficient protein degradation functions after oral administration. The work first applied PROTACs as a chemical knockdown technology to achieve efficient degradation of the target protein in animals, which provided a powerful tool for the research of target protein depletion in adult animals.

In addition, the Trauner group first developed a series of novel optical controlled PROTACs, named PHOTACs, for targeting FKBP12 with an azobenzene photoswitch, which inserted in the linker part of dFKBP-1 for proof of concept^338^ (Fig. 48). The synthesized PHOTAC-II-5 showed significant degradation of FKBP12 between the concentrations of 10 nM and 3 µM under irradiation with a wavelength of 390 nM in RS4;11 cells. The PHOTAC approach provided a new direction for photomedicine, which can precisely regulate the degradation of target proteins through optical control.

## Conclusion and perspectives

Though the emergence of kinase inhibitors, molecularly targeted therapy and immunotherapy have brought a bright future for patients, although many tough questions remain. The major problem is the ever-increasing drug resistance after receiving different kinds of therapy, including intrinsic and adaptive resistance. Only 20–25% of all protein targets are currently being studied, which means that the remaining targets are still unexplored. How to make more potential drug targets accessible becomes another serious question for researchers. Beyond that, the focus of the current exploration of the targets is the enzymatic functions, while protein function also depends on their scaffold. In addition, the selectivity of classic inhibitors is poor. As a novel and powerful strategy, PROTACs have attracted great attention both from academia and industry for discovering new types of therapeutic agents relying on their unique characteristic of degrading target proteins instead of inhibiting them. Therefore, PROTACs are particularly sensitive to drug-resistant targets, which are commonly caused by exposure to high concentrations of small molecule inhibitors. Since PROTACs act catalytically, the scope of proteins used for treating diseases has been expanded by PROTACs. In addition, PROTACs exhibited better selectivity when compared to classic inhibitors. More importantly, PROTACs can affect the nonenzymatic functions of proteins, facilitating the control of protein functions that are not easily achieved by traditional small-molecule therapeutics. In addition, PROTACs present a chemical knockdown approach with speed and reversibility. Unlike other gene editing tools, PROTACs are quick and direct at the protein level, which can avoid misinterpretations arising from potential genetic compensation and/or spontaneous mutations. Thus, PROTACs have been widely explored around the world with degraders of 42 targets published. These degraders not only outperformed in cancer diseases but also in immune disorders, viral infections and neurodegenerative diseases.

Conclusively, PROTACs present a very promising and powerful approach for crossing the hurdle of present drug discovery and tool development in biology. On the other hand, more efforts are needed to gain deep insight into the efficacy and safety of PROTACs in the clinic. More target binders and more E3 ligases applicable in the development of PROTACs are waiting for exploration.

## References

[1] Toure M, Crews CM (2016). Small-molecule PROTACS: new approaches to protein degradation. Angew. Chem. Int Ed. Engl..

[2] Zou Y, Ma D, Wang Y, Zou Y (2019). The PROTAC technology in drug development. Cell Biochem. Funct..

[3] Yang C-Y, Qin C, Bai L, Wang S (2019). Small-molecule PROTAC degraders of the Bromodomain and Extra Terminal (BET) proteins - A review. Drug Discov. Today Technol..

[4] Wurz RP, Cee VJ (2019). Targeted degradation of MDM2 as a new approach to improve the efficacy of MDM2-p53 inhibitors. J. Med. Chem..

[5] Watt GF, Scott-Stevens P, Gaohua L (2019). Targeted protein degradation in vivo with proteolysis targeting chimeras: current status and future considerations. Drug Discov. Today Technol..

[6] Wang P, Zhou J (2018). Proteolysis targeting chimera (PROTAC): a paradigm-shifting approach in small molecule drug discovery. Curr. Top. Med. Chem..

[7] Walczak MJ, Petzold G, Thoma NH (2017). Targeted protein degradation. You can glue it too!. Nat. Chem. Biol..

[8] Veggiani G, Gerpe MCR, Sidhu SS, Zhang W (2019). Emerging drug development technologies targeting ubiquitination for cancer therapeutics. Pharm. Ther..

[9] Tan L, Gray NS (2018). When kinases meet PROTACs. Chin. J. Chem..

[10] Scheepstra M, Hekking KFW, van Hijfte L, Folmer RHA (2019). Bivalent ligands for protein degradation in drug discovery. Comput. Struct. Biotechnol. J..

[11] Sakamoto KM (2010). Protacs for treatment of cancer. Pediatr. Res..

[12] Sakamoto KM (2005). Chimeric molecules to target proteins for ubiquitination and degradation. Methods Enzymol..

[13] Runcie AC, Chan K-H, Zengerle M, Ciulli A (2016). Chemical genetics approaches for selective intervention in epigenetics. Curr. Opin. Chem. Biol..

[14] Roth S, Fulcher LJ, Sapkota GP (2019). Advances in targeted degradation of endogenous proteins. Cell Mol. Life Sci..

[15] Raina K, Crews CM (2010). Chemical inducers of targeted protein degradation. J. Biol. Chem..

[16] Pettersson M, Crews CM (2019). PROteolysis TArgeting Chimeras (PROTACs) - Past, present and future. Drug Discov. Today Technol..

[17] Paiva S-L, Crews CM (2019). Targeted protein degradation: elements of PROTAC design. Curr. Opin. Chem. Biol..

[18] Ottis P, Crews CM (2017). Proteolysis-targeting chimeras: induced protein degradation as a therapeutic strategy. ACS Chem. Biol..

[19] Ohoka N (2018). Development of protein knockdown technology as emerging drug discovery strategy. Yakugaku Zasshi..

[20] Nguyen C, West GM, Geoghegan KF (2017). Emerging methods in chemoproteomics with relevance to drug discovery. Methods Mol. Biol..

[21] Neklesa TK, Winkler JD, Crews CM (2017). Targeted protein degradation by PROTACs. Pharmacol. Ther..

[22] Moon S, Lee B-H (2018). Chemically induced cellular proteolysis: an emerging therapeutic strategy for undruggable targets. Mol. Cells.

[23] Mansour MA (2018). Ubiquitination: friend and foe in cancer. Int J. Biochem. Cell Biol..

[24] Lebraud H, Heightman TD (2017). Protein degradation: a validated therapeutic strategy with exciting prospects. Essays Biochem..

[25] Kong, X. et al. Drug discovery targeting anaplastic lymphoma kinase (ALK). *J. Med. Chem*. 10.1021/acs.jmedchem.9b00446 (2019). 10.1021/acs.jmedchem.9b00446 31419130

[26] Koh DCI, Armugam A, Jeyaseelan K (2006). Snake venom components and their applications in biomedicine. Cell. Mol. Life Sci..

[27] Jones LH (2018). Small-molecule kinase downregulators. Cell Chem. Biol..

[28] Hughes SJ, Ciulli A (2017). Molecular recognition of ternary complexes: a new dimension in the structure-guided design of chemical degraders. Essays Biochem..

[29] Gu S (2018). PROTACs: an emerging targeting technique for protein degradation in drug discovery. Bioessays.

[30] Gao N (2017). Chemical methods to knock down the amyloid proteins. Molecules.

[31] Flanagan JJ, Neklesa TK (2019). Targeting nuclear receptors with PROTAC degraders. Mol. Cell. Endocrinol..

[32] Edmondson SD, Yang B, Fallan C (2019). Proteolysis targeting chimeras (PROTACs) in ‘beyond rule-of-five' chemical space: recent progress and future challenges. Bioorg. Med. Chem. Lett..

[33] Deshaies RJ (2015). Protein degradation. Prime time for PROTACs. Nat. Chem. Biol..

[34] Daniels DL, Riching KM, Urh M (2019). Monitoring and deciphering protein degradation pathways inside cells. Drug Discov. Today Technol..

[35] Churcher I (2018). Protac-induced protein degradation in drug discovery: breaking the rules or just making new ones?. J. Med. Chem..

[36] Cheng J (2019). The emerging role for Cullin 4 family of E3 ligases in tumorigenesis. Biochim. Biophys. Acta, Rev. Cancer.

[37] Cermakova K, Hodges HC, Hodges HC (2018). Next-generation drugs and probes for chromatin biology: from targeted protein degradation to phase separation. Molecules.

[38] Buhimschi AD, Crews CM (2019). Evolving rules for protein degradation? Insights from the zinc finger degrome. Biochemistry.

[39] Asmat A, Ramzan F (2018). Venom protein C activators as diagnostic agents for defects of protein C system. Protein Pept. Lett..

[40] Kang CH (2018). Induced protein degradation of anaplastic lymphoma kinase (ALK) by proteolysis targeting chimera (PROTAC). Biochem Biophys. Res. Commun..

[41] Konstantinidou M (2019). PROTACs- a game-changing technology. Expert Opin. Drug Discov..

[42] Roy MJ (2019). SPR-measured dissociation kinetics of PROTAC ternary complexes influence target degradation rate. ACS Chem. Biol..

[43] Riching KM (2018). Quantitative live-cell kinetic degradation and mechanistic profiling of PROTAC mode of action. ACS Chem. Biol..

[44] An S, Fu L (2018). Small-molecule PROTACs: an emerging and promising approach for the development of targeted therapy drugs. EBioMedicine.

[45] Drummond ML, Williams CI (2019). In silico modeling of PROTAC-mediated ternary complexes: validation and application. J. Chem. Inf. Model..

[46] Farnaby W (2019). BAF complex vulnerabilities in cancer demonstrated via structure-based PROTAC design. Nat. Chem. Biol..

[47] Smith BE (2019). Differential PROTAC substrate specificity dictated by orientation of recruited E3 ligase. Nat. Commun..

[48] Nowak RP (2018). Plasticity in binding confers selectivity in ligand-induced protein degradation. Nat. Chem. Biol..

[49] Gadd MS (2017). Structural basis of PROTAC cooperative recognition for selective protein degradation. Nat. Chem. Biol..

[50] Yang Jiuling, Li Yangbing, Aguilar Angelo, Liu Zhaomin, Yang Chao-Yie, Wang Shaomeng (2019). Simple Structural Modifications Converting a Bona fide MDM2 PROTAC Degrader into a Molecular Glue Molecule: A Cautionary Tale in the Design of PROTAC Degraders. Journal of Medicinal Chemistry.

[51] Banik, S. M. et al. Lysosome targeting chimeras (LYTACs) for the degradation of secreted and membrane proteins. *ChemRxiv*, 1–68 (2019).

[52] Reynders, M. et al. PHOTACs enable optical control of protein degradation. *ChemRxiv*, 1–34 (2019). 10.1007/978-1-0716-1665-9_17 34432252

[53] Naro, Y., Darrah, K. & Deiters, A. Optical control of small molecule-induced protein degradation. *ChemRxiv*, 1–9 (2019). 10.1021/jacs.9b12718 PMC7229639 31927988

[54] Xue G (2019). Light-induced protein degradation with photocaged PROTACs. J. Am. Chem. Soc..

[55] Pfaff P, Samarasinghe KTG, Crews CM, Carreira EM (2019). Reversible spatiotemporal control of induced protein degradation by bistable photoPROTACs. ACS Cent. Sci..

[56] Takahashi D (2019). AUTACs: cargo-specific degraders using selective autophagy. Mol. Cell.

[57] Maniaci C (2017). Homo-PROTACs: bivalent small-molecule dimerizers of the VHL E3 ubiquitin ligase to induce self-degradation. Nat. Commun..

[58] Steinebach C (2018). Homo-PROTACs for the chemical knockdown of cereblon. ACS Chem. Biol..

[59] Girardini M (2019). Cereblon versus VHL: hijacking E3 ligases against each other using PROTACs. Bioorgan. Med. Chem..

[60] Steinebach C (2019). PROTAC-mediated crosstalk between E3 ligases. Chem. Commun..

[61] Li YB (2019). Discovery of MD-224 as a first-in-class, highly potent, and efficacious proteolysis targeting chimera murine double minute 2 degrader capable of achieving complete and durable tumor regression. J. Med. Chem..

[62] Lebraud H, Wright DJ, Johnson CN, Heightman TD (2016). Protein degradation by in-cell self-assembly of proteolysis targeting chimeras. ACS Cent. Sci..

[63] Schneekloth AR, Pucheault M, Tae HS, Crews CM (2008). Targeted intracellular protein degradation induced by a small molecule: en route to chemical proteomics. Bioorg. Med Chem. Lett..

[64] Vassilev LT (2004). In vivo activation of the p53 pathway by small-molecule antagonists of MDM2. Science.

[65] Itoh Y, Ishikawa M, Naito M, Hashimoto Y (2010). Protein knockdown using methyl bestatin-ligand hybrid molecules: design and synthesis of inducers of ubiquitination-mediated degradation of cellular retinoic acid-binding proteins. J. Am. Chem. Soc..

[66] Shibata N (2018). Development of protein degradation inducers of androgen receptor by conjugation of androgen receptor ligands and inhibitor of apoptosis protein ligands. J. Med. Chem..

[67] Ohoka N (2019). Different degradation mechanisms of inhibitor of apoptosis proteins (IAPs) by the specific and nongenetic IAP-dependent protein eraser (SNIPER). Chem. Pharm. Bull..

[68] Ohoka, N. et al. Development of small molecule chimeras that recruit AhR E3 ligase to target proteins. *ACS Chem. Biol*. (2019). 10.1021/acschembio.9b00704 31580635

[69] Ohoka N, Shibata N, Hattori T, Naito M (2016). Protein knockdown technology: application of ubiquitin ligase to cancer therapy. Curr. Cancer Drug Targets.

[70] Ohoka N (2017). In vivo knockdown of pathogenic proteins via specific and nongenetic inhibitor of apoptosis protein (IAP)-dependent protein erasers (SNIPERs). J. Biol. Chem..

[71] Ohoka N (2018). Derivatization of inhibitor of apoptosis protein (IAP) ligands yields improved inducers of estrogen receptor α degradation. J. Biol. Chem..

[72] Ohoka N (2017). Development of a peptide-based inducer of protein degradation targeting NOTCH1. Bioorg. Med. Chem. Lett..

[73] Naito M, Ohoka N, Shibata N, SNIPERs-Hijacking IAP (2019). SNIPERs-hijacking IAP activity to induce protein degradation. Drug Discov. Today Technol..

[74] Maple HJ (2019). Developing degraders: principles and perspectives on design and chemical space. MedChemComm.

[75] Itoh Y (2011). Design, synthesis and biological evaluation of nuclear receptor-degradation inducers. Bioorg. Med. Chem..

[76] Itoh Y (2011). Development of target protein-selective degradation inducer for protein knockdown. Bioorg. Med. Chem..

[77] Buckley DL (2012). Targeting the von Hippel-Lindau E3 ubiquitin ligase using small molecules to disrupt the VHL/HIF-1alpha interaction. J. Am. Chem. Soc..

[78] Van Molle I (2012). Dissecting fragment-based lead discovery at the von Hippel-Lindau protein:hypoxia inducible factor 1alpha protein-protein interface. Chem. Biol..

[79] Testa A (2018). 3-Fluoro-4-hydroxyprolines: synthesis, conformational analysis, and stereoselective recognition by the VHL E3 ubiquitin ligase for targeted protein degradation. J. Am. Chem. Soc..

[80] Lopez-Girona A (2012). Cereblon is a direct protein target for immunomodulatory and antiproliferative activities of lenalidomide and pomalidomide. Leukemia.

[81] Kronke J (2014). Lenalidomide causes selective degradation of IKZF1 and IKZF3 in multiple myeloma cells. Science.

[82] Lu J (2015). Hijacking the E3 ubiquitin ligase cereblon to efficiently target BRD4. Chem. Biol..

[83] Marin JJ (2012). Chemoprevention, chemotherapy, and chemoresistance in colorectal cancer. Drug Metab. Rev..

[84] Holohan C, Van Schaeybroeck S, Longley DB, Johnston PG (2013). Cancer drug resistance: an evolving paradigm. Nat. Rev. Cancer.

[85] Lohitesh K, Chowdhury R, Mukherjee S (2018). Resistance a major hindrance to chemotherapy in hepatocellular carcinoma: an insight. Cancer Cell Int..

[86] Vimalavathini R, Sindhuja A, Sreemathy K, Jagadesan R (2018). Strategies to overcome chemotherapeutic drug resistance - a mini review. World J. Pharm. Res..

[87] Wu P, Nielsen TE, Clausen MH (2015). FDA-approved small-molecule kinase inhibitors. Trends Pharm. Sci..

[88] Ozvegy-Laczka C, Cserepes J, Elkind NB, Sarkadi B (2005). Tyrosine kinase inhibitor resistance in cancer: role of ABC multidrug transporters. Drug Resist Updat..

[89] Babina IS, Turner NC (2017). Advances and challenges in targeting FGFR signalling in cancer. Nat. Rev. Cancer.

[90] Dong L, Lei D, Zhang H (2017). Clinical strategies for acquired epidermal growth factor receptor tyrosine kinase inhibitor resistance in non-small-cell lung cancer patients. Oncotarget.

[91] Gounder MM, Maki RG (2011). Molecular basis for primary and secondary tyrosine kinase inhibitor resistance in gastrointestinal stromal tumor. Cancer Chemother. Pharmacol..

[92] Camidge DR, Pao W, Sequist LV (2014). Acquired resistance to TKIs in solid tumours: learning from lung cancer. Nat. Rev. Clin. Oncol..

[93] Francica P, Rottenberg S (2018). Mechanisms of PARP inhibitor resistance in cancer and insights into the DNA damage response. Genome Med..

[94] Buschbeck M (2006). Strategies to overcome resistance to targeted protein kinase inhibitors in the treatment of cancer. Drugs R. D..

[95] Oxnard GR (2011). New strategies in overcoming acquired resistance to epidermal growth factor receptor tyrosine kinase inhibitors in lung cancer. Clin. Cancer Res..

[96] Matsiko A (2018). Cancer immunotherapy making headway. Nat. Mater..

[97] Jackson CM, Choi J, Lim M (2019). Mechanisms of immunotherapy resistance: lessons from glioblastoma. Nat. Immunol..

[98] Redell MS (2016). A STAT3 decoy lures AML out of hiding. Blood.

[99] Jinesh GG (2018). Molecular genetics and cellular events of K-Ras-driven tumorigenesis. Oncogene.

[100] Cromm PM, Samarasinghe KTG, Hines J, Crews CM (2018). Addressing kinase-independent functions of fak via PROTAC-mediated degradation. J. Am. Chem. Soc..

[101] Wang S (2018). Design and synthesis of proteolysis targeting chimeras for inducing BRD4 protein degradation. Chem. Res. Chin. Universities..

[102] Cyrus K (2011). Impact of linker length on the activity of PROTACs. Mol. Biosyst..

[103] Bondeson DP (2018). Lessons in PROTAC design from selective degradation with a promiscuous warhead. Cell Chem. Biol..

[104] Zhang L, Riley-Gillis B, Vijay P, Shen Y (2019). Acquired resistance to BET-PROTACs (proteolysis-targeting chimeras) caused by genomic alterations in core components of E3 ligase complexes. Mol. Cancer Ther..

[105] Matyskiela ME (2016). A novel cereblon modulator recruits GSPT1 to the CRL4(CRBN) ubiquitin ligase. Nature.

[106] Bock KW, Kohle C (2005). Ah receptor- and TCDD-mediated liver tumor promotion: clonal selection and expansion of cells evading growth arrest and apoptosis. Biochem Pharmacol..

[107] Swanson HI (2002). DNA binding and protein interactions of the AHR/ARNT heterodimer that facilitate gene activation. Chem.-Biol. Interact..

[108] Lee H (2007). Targeted degradation of the aryl hydrocarbon receptor by the PROTAC approach: a useful chemical genetic tool. Chembiochem.

[109] Morris SW (1995). Fusion of a kinase gene, ALK, to a nucleolar protein gene, NPM, in non-Hodgkin's lymphoma. Science.

[110] De Paepe P (2003). ALK activation by the CLTC-ALK fusion is a recurrent event in large B-cell lymphoma. Blood.

[111] Soda M (2007). Identification of the transforming EML4-ALK fusion gene in non-small-cell lung cancer. Nature.

[112] Debelenko LV (2011). Renal cell carcinoma with novel VCL-ALK fusion: new representative of ALK-associated tumor spectrum. Mod. Pathol..

[113] Chen Y (2008). Oncogenic mutations of ALK kinase in neuroblastoma. Nature.

[114] Kelly LM (2014). Identification of the transforming STRN-ALK fusion as a potential therapeutic target in the aggressive forms of thyroid cancer. Proc. Natl Acad. Sci. USA.

[115] Ren H (2012). Identification of anaplastic lymphoma kinase as a potential therapeutic target in ovarian cancer. Cancer Res..

[116] Bayliss R (2016). Molecular mechanisms that underpin EML4-ALK driven cancers and their response to targeted drugs. Cell Mol. Life Sci..

[117] George RE (2008). Activating mutations in ALK provide a therapeutic target in neuroblastoma. Nature.

[118] Passoni L (2009). Mutation-independent anaplastic lymphoma kinase overexpression in poor prognosis neuroblastoma patients. Cancer Res..

[119] Salido M (2011). Increased ALK gene copy number and amplification are frequent in non-small cell lung cancer. J. Thorac. Oncol..

[120] Lin JJ, Riely GJ, Shaw AT (2017). Targeting ALK: precision medicine takes on drug resistance. Cancer Discov..

[121] Choi YL (2010). EML4-ALK mutations in lung cancer that confer resistance to ALK inhibitors. N. Engl. J. Med..

[122] Powell CE (2018). Chemically induced degradation of anaplastic lymphoma kinase (ALK). J. Med Chem..

[123] Zhang C (2018). Proteolysis targeting chimeras (PROTACs) of Anaplastic Lymphoma Kinase (ALK). Eur. J. Med Chem..

[124] Miyamoto H, Altuwaijri S, Chang C (2004). Androgen receptor in prostate cancer progression. Endocr. Rev..

[125] Barbara K, Joanne C, Natasha K (2014). Androgen receptor as a driver of therapeutic resistance in advanced prostate cancer. Int. J. Biol. Sci..

[126] Salami J (2018). Androgen receptor degradation by the proteolysis-targeting chimera ARCC-4 outperforms enzalutamide in cellular models of prostate cancer drug resistance. Commun. Biol..

[127] Pentimalli Francesca (2017). BCL2: a 30-year tale of life, death and much more to come. Cell Death & Differentiation.

[128] Wang Z (2019). Proteolysis Targeting Chimeras for the Selective Degradation of Mcl-1/Bcl-2 Derived from Nonselective Target Binding Ligands. J. Med. Chem..

[129] Ennishi D (2017). Genetic profiling of MYC and BCL2 in diffuse large B-cell lymphoma determines cell-of-origin–specific clinical impact. Blood.

[130] Burkhard R (2015). BCL2 mutation spectrum in B-cell non-Hodgkin lymphomas and patterns associated with evolution of follicular lymphoma. Hematol. Oncol..

[131] Huynh KD, Verdin FW, Bardwell E (2000). VJ. BCoR, a novel corepressor involved in BCL-6 repression. Genes Dev..

[132] Nurieva RI (2009). Bcl6 mediates the development of T follicular helper cells. Science.

[133] Dent AL (1997). Control of inflammation, cytokine expression, and germinal center formation by BCL-6. Science.

[134] Pasqualucci L, Dalla-Favera R (2014). SnapShot: diffuse large B cell lymphoma. Cancer Cell.

[135] Basso K, Dalla-Favera R (2012). Roles of BCL6 in normal and transformed germinal center B cells. Immunol. Rev..

[136] Cardenas MG (2017). The expanding role of the BCL6 oncoprotein as a cancer therapeutic target. Clin. Cancer Res..

[137] Cardenas MG (2016). Rationally designed BCL6 inhibitors target activated B cell diffuse large B cell lymphoma. J. Clin. Invest..

[138] Cerchietti LC (2009). A peptomimetic inhibitor of BCL6 with potent antilymphoma effects in vitro and in vivo. Blood.

[139] McCoull W (2018). Development of a novel B-cell lymphoma 6 (BCL6) PROTAC to provide insight into small molecule targeting of BCL6. ACS Chem. Biol..

[140] Hantschel O, Superti-Furga G (2004). Regulation of the c-Abl and Bcr-Abl tyrosine kinases. Nat. Rev. Mol. Cell Biol..

[141] Hantschel O (2012). BCR-ABL uncouples canonical JAK2-STAT5 signaling in chronic myeloid leukemia. Nat. Chem. Biol..

[142] Druker BJ (1996). Effects of a selective inhibitor of the Abl tyrosine kinase on the growth of Bcr–Abl positive cells. Nat. Med..

[143] Schittenhelm MM (2006). Dasatinib (BMS-354825), a dual SRC/ABL kinase inhibitor, inhibits the kinase activity of wild-type, juxtamembrane, and activation loop mutant KIT isoforms associated with human malignancies. Cancer Res..

[144] Weisberg E (2006). AMN107 (nilotinib): a novel and selective inhibitor of BCR-ABL. Br. J. Cancer.

[145] Huang W-S (2010). Discovery of 3-[2-(Imidazo[1,2-b]pyridazin-3-yl)ethynyl]-4-methyl-N-{4-[(4-methylpiperazin-1-yl)methyl]-3-(trifluoromethyl)phenyl}benzamide (AP24534), a Potent, Orally Active Pan-Inhibitor of Breakpoint Cluster Region-Abelson (BCR-ABL) kinase including the T315I gatekeeper mutant. J. Med. Chem..

[146] Lai AC (2016). Modular PROTAC design for the degradation of oncogenic BCR-ABL. Angew. Chem. Int Ed. Engl..

[147] Shimokawa K (2017). Targeting the allosteric site of oncoprotein BCR-ABL as an alternative strategy for effective target protein degradation. ACS Med. Chem. Lett..

[148] Mottet N (2011). EAU Guidelines on Prostate Cancer. Part II: Treatment of Advanced, Relapsing, and Castration-Resistant Prostate Cancer. Eur. Urol..

[149] Antonarakis ES (2014). AR-V7 and resistance to enzalutamide and abiraterone in prostate cancer. N. Engl. J. Med..

[150] Anastasia W (2013). Inhibition of BET bromodomain proteins as a therapeutic approach in prostate cancer. Oncotarget.

[151] Winter GE (2015). Phthalimide conjugation as a strategy for in vivo target protein degradation. Science.

[152] Raina K (2016). PROTAC-induced BET protein degradation as a therapy for castration-resistant prostate cancer. Proc. Natl Acad. Sci. USA.

[153] Bai L (2017). Targeted degradation of BET proteins in triple-negative breast cancer. Cancer Res..

[154] Zengerle M, Chan K-H, Ciulli A (2015). Selective small molecule induced degradation of the BET bromodomain protein BRD4. ACS Chem. Biol..

[155] Zhou B (2017). Discovery of a small-molecule degrader of bromodomain and extra-terminal (BET) proteins with picomolar cellular potencies and capable of achieving tumor regression. J. Med. Chem..

[156] Qin C (2018). Discovery of QCA570 as an exceptionally potent and efficacious proteolysis targeting chimera (PROTAC) degrader of the bromodomain and extra-terminal (BET) proteins capable of inducing complete and durable tumor regression. J. Med. Chem..

[157] Kadoch C (2013). Proteomic and bioinformatic analysis of mammalian SWI/SNF complexes identifies extensive roles in human malignancy. Nat. Genet..

[158] Kaeser M (2008). BRD7, a novel PBAF-specific SWI/SNF Subunit, is required for target gene activation and repression in embryonic stem cells. J. Biol. Chem..

[159] Hohmann AF, Vakoc CR (2014). A rationale to target the SWI/SNF complex for cancer therapy. Trends Genet..

[160] Scotto L (2008). Integrative genomics analysis of chromosome 5p gain in cervical cancer reveals target over-expressed genes, including Drosha. Mol. Cancer.

[161] Remillard D (2017). Degradation of the BAF complex factor BRD9 by heterobifunctional ligands. Angew. Chem. Int. Ed..

[162] Zoppi V (2019). Iterative design and optimization of initially inactive proteolysis targeting chimeras (PROTACs) identify VZ185 as a potent, fast, and selective von Hippel-Lindau (VHL) based dual degrader probe of BRD9 and BRD7. J. Med. Chem..

[163] Mohamed AJ (2010). Bruton's tyrosine kinase (Btk): function, regulation, and transformation with special emphasis on the PH domain. Immunological Rev..

[164] Pan Z (2007). Discovery of selective irreversible inhibitors for Bruton's tyrosine kinase. ChemMedChem.

[165] Woyach JA (2014). Resistance mechanisms for the Bruton's tyrosine kinase inhibitor ibrutinib. N. Engl. J. Med..

[166] Sun Y (2018). PROTAC-induced BTK degradation as a novel therapy for mutated BTK C481S induced ibrutinib-resistant B-cell malignancies. Cell Res..

[167] Sun Y (2019). Degradation of Bruton’s tyrosine kinase mutants by PROTACs for potential treatment of ibrutinib-resistant non-Hodgkin lymphomas. Leukemia.

[168] Buhimschi AD (2018). Targeting the C481S ibrutinib-resistance mutation in Bruton's tyrosine kinase using PROTAC-mediated degradation. Biochemistry.

[169] Huang HT (2018). A chemoproteomic approach to query the degradable kinome using a multi-kinase degrader. Cell Chem. Biol..

[170] Zorba A (2018). Delineating the role of cooperativity in the design of potent PROTACs for BTK. Proc. Natl Acad. Sci. USA.

[171] Zhao B, Burgess K (2019). PROTACs suppression of CDK4/6, crucial kinases for cell cycle regulation in cancer. Chem. Commun..

[172] Jiang B (2019). Development of dual and selective degraders of cyclin-dependent kinases 4 and 6. Angew. Chem. Int Ed. Engl..

[173] Brand M (2019). Homolog-selective degradation as a strategy to probe the function of CDK6 in AML. Cell Chem. Biol..

[174] Su S (2019). Potent and preferential degradation of CDK6 via proteolysis targeting chimera degraders. J. Med. Chem..

[175] Garrido-Castro AC, Goel S (2017). CDK4/6 inhibition in breast cancer: mechanisms of response and treatment failure. Curr. Breast Cancer Rep..

[176] Yang C (2016). Acquired CDK6 amplification promotes breast cancer resistance to CDK4/6 inhibitors and loss of ER signaling and dependence. Oncogene.

[177] Allen BL, Taatjes DJ (2015). The Mediator complex: a central integrator of transcription. Nat. Rev. Mol. Cell Biol..

[178] Carlsten JO, Zhu X, Gustafsson CM (2013). The multitalented mediator complex. Trends Biochem. Sci..

[179] Schiano C (2014). Involvement of mediator complex in malignancy. Biochim Biophys. Acta.

[180] Firestein R (2008). CDK8 is a colorectal cancer oncogene that regulates beta-catenin activity. Nature.

[181] Rzymski T, Mikula M, Wiklik K, Brzozka K (2015). CDK8 kinase–An emerging target in targeted cancer therapy. Biochim Biophys. Acta.

[182] Hatcher JM (2018). Development of highly potent and selective steroidal inhibitors and degraders of CDK8. ACS Med. Chem. Lett..

[183] Sonawane YA (2016). Cyclin dependent kinase 9 inhibitors for cancer therapy. J. Med. Chem..

[184] Simone C, Giordano A (2007). Abrogation of signal-dependent activation of the cdk9/cyclin T2a complex in human RD rhabdomyosarcoma cells. Cell Death Differ..

[185] de Falco G, Giordano A (1998). CDK9 (PITALRE): a multifunctional cdc2-related kinase. J. Cell Physiol..

[186] Bagella L (1998). Cloning of murine CDK9/PITALRE and its tissue-specific expression in development. J. Cell Physiol..

[187] Gregory GP (2015). CDK9 inhibition by dinaciclib potently suppresses Mcl-1 to induce durable apoptotic responses in aggressive MYC-driven B-cell lymphoma in vivo. Leukemia.

[188] Yeh YY (2015). Up-regulation of CDK9 kinase activity and Mcl-1 stability contributes to the acquired resistance to cyclin-dependent kinase inhibitors in leukemia. Oncotarget.

[189] Krystof V, Uldrijan S (2010). Cyclin-dependent kinase inhibitors as anticancer drugs. Curr. Drug Targets.

[190] Petzold G, Fischer ES, Thoma NH (2016). Structural basis of lenalidomide-induced CK1alpha degradation by the CRL4(CRBN) ubiquitin ligase. Nature.

[191] Robb CM (2017). Chemically induced degradation of CDK9 by a proteolysis targeting chimera (PROTAC). Chem. Commun..

[192] Olson CM (2018). Pharmacological perturbation of CDK9 using selective CDK9 inhibition or degradation. Nat. Chem. Biol..

[193] Bian J (2018). Discovery of Wogonin-based PROTACs against CDK9 and capable of achieving antitumor activity. Bioorg. Chem..

[194] Litchfield DW (2003). Protein kinase CK2: structure, regulation and role in cellular decisions of life and death. Biochem. J..

[195] Landesman-Bollag E (2001). Protein kinase CK2 in mammary gland tumorigenesis. Oncogene.

[196] Chen H (2018). Chemically induced degradation of CK2 by proteolysis targeting chimeras based on a ubiquitin-proteasome pathway. Bioorg. Chem..

[197] Organ ST, Tsao MS (2011). An overview of the c-MET signaling pathway. Therapeutic Adv. Med. Oncol..

[198] Burslem GM (2018). The advantages of targeted protein degradation over inhibition: an RTK case study. Cell Chem. Biol..

[199] Yang Y (2019). Discovery, optimization, and target identification of novel potent broad-spectrum antiviral inhibitors. J. Med. Chem..

[200] Madak JT (2017). Design, synthesis, and characterization of brequinar conjugates as probes to study DHODH inhibition. Chemistry.

[201] Takeuchi K, Ito F (2011). Receptor tyrosine kinases and targeted cancer therapeutics. Biol. Pharm. Bull..

[202] Lemmon MA, Schlessinger J (2010). Cell signaling by receptor tyrosine kinases. Cell.

[203] Engelman JA (2007). MET amplification leads to gefitinib resistance in lung cancer by activating ERBB3 signaling. Science.

[204] Graves LM, Duncan JS, Whittle MC, Johnson GL (2013). The dynamic nature of the kinome. Biochem J..

[205] Bondeson DP (2015). Catalytic in vivo protein knockdown by small-molecule PROTACs. Nat. Chem. Biol..

[206] Sakamoto KM (2001). Protacs: chimeric molecules that target proteins to the Skp1-Cullin-F box complex for ubiquitination and degradation. Proc. Natl Acad. Sci. USA.

[207] Sonenberg N, Hinnebusch AG (2009). Regulation of translation initiation in eukaryotes: mechanisms and biological targets. Cell.

[208] Mamane Y (2004). eIF4E–from translation to transformation. Oncogene.

[209] Jia Y, Polunovsky V, Bitterman PB, Wagner CR (2012). Cap-dependent translation initiation factor eIF4E: an emerging anticancer drug target. Med. Res. Rev..

[210] Avdulov S (2004). Activation of translation complex eIF4F is essential for the genesis and maintenance of the malignant phenotype in human mammary epithelial cells. Cancer Cell..

[211] Kerekatte V (1995). The proto-oncogene/translation factor eIF4E: a survey of its expression in breast carcinomas. Int. J. Cancer.

[212] Graff JR (2007). Therapeutic suppression of translation initiation factor eIF4E expression reduces tumor growth without toxicity. J. Clin. Invest..

[213] Brown CJ, McNae I, Fischer PM, Walkinshaw MD (2007). Crystallographic and mass spectrometric characterisation of eIF4E with N7-alkylated cap derivatives. J. Mol. Biol..

[214] Fischer PM (2009). Cap in hand: targeting eIF4E. Cell Cycle.

[215] Kaur T, Menon A, Garner AL (2019). Synthesis of 7-benzylguanosine cap-analogue conjugates for eIF4E targeted degradation. Eur. J. Med. Chem..

[216] Stefan N, Koehler KF, Jan-Ke G (2011). Development of subtype-selective oestrogen receptor-based therapeutics. Nat. Rev. Drug Discov..

[217] Hu J (2019). Discovery of ERD-308 as a highly potent proteolysis targeting chimera (PROTAC) degrader of estrogen receptor (ER). J. Med. Chem..

[218] Samatar AA, Poulikakos PI (2014). Targeting RAS-ERK signalling in cancer: promises and challenges. Nat. Rev. Drug Discov..

[219] Roskoski R (2012). ERK1/2 MAP kinases: structure, function, and regulation. Pharm. Res..

[220] Giguere V, Yang N, Segui P, Evans RM (1988). Identification of a new class of steroid hormone receptors. Nature.

[221] Eudy JD (1998). Isolation of a gene encoding a novel member of the nuclear receptor superfamily from the critical region of usher syndrome type IIa at 1q41. Genomics.

[222] Luo J (1997). Placental abnormalities in mouse embryos lacking the orphan nuclear receptor ERR-beta. Nature.

[223] Audet-Walsh E, Giguere V (2015). The multiple universes of estrogen-related receptor alpha and gamma in metabolic control and related diseases. Acta Pharm. Sin..

[224] Puigserver P, Spiegelman BM (2003). Peroxisome proliferator-activated receptor-gamma coactivator 1 alpha (PGC-1 alpha): transcriptional coactivator and metabolic regulator. Endocr. Rev..

[225] Christian M, White R, Parker MG (2006). Metabolic regulation by the nuclear receptor corepressor RIP140. Trends Endocrinol. Metab..

[226] Peng L (2019). Identification of new small-molecule inducers of estrogen-related receptor alpha (ERRalpha) degradation. ACS Med. Chem. Lett..

[227] van Nimwegen MJ, van de Water B (2007). Focal adhesion kinase: a potential target in cancer therapy. Biochem. Pharmacol..

[228] Kessler BE (2016). FAK expression, not kinase activity, is a key mediator of thyroid tumorigenesis and protumorigenic processes. Mol. Cancer Res..

[229] Beraud C (2015). Targeting FAK scaffold functions inhibits human renal cell carcinoma growth. Int. J. Cancer.

[230] Gogate PN (2014). Targeting the C-terminal focal adhesion kinase scaffold in pancreatic cancer. Cancer Lett..

[231] Gungor-Ordueri NE (2014). New insights into FAK function and regulation during spermatogenesis. Histol. Histopathol..

[232] Schaller MD (2010). Cellular functions of FAK kinases: insight into molecular mechanisms and novel functions. J. Cell Sci..

[233] Li SY, Mruk DD, Cheng CY (2013). Focal adhesion kinase is a regulator of F-actin dynamics: new insights from studies in the testis. Spermatogenesis.

[234] Hall JE, Fu W, Schaller MD (2011). Focal adhesion kinase: exploring Fak structure to gain insight into function. Int Rev. Cell Mol. Biol..

[235] Jung Y, McCarty JH (2012). Band 4.1 proteins regulate integrin-dependent cell spreading. Biochem. Biophys. Res. Commun..

[236] Lim ST (2012). Nuclear-localized focal adhesion kinase regulates inflammatory VCAM-1 expression. J. Cell. Biol..

[237] Frame MC (2010). The FERM domain: organizing the structure and function of FAK. Nat. Rev. Mol. Cell. Biol..

[238] Brami-Cherrier K (2014). FAK dimerization controls its kinase-dependent functions at focal adhesions. EMBO J..

[239] Lee BY, Timpson P, Horvath LG, Daly RJ (2015). FAK signaling in human cancer as a target for therapeutics. Pharm. Ther..

[240] Mitra SK, Hanson DA, Schlaepfer DD (2005). Focal adhesion kinase: in command and control of cell motility. Nat. Rev. Mol. Cell. Biol..

[241] Roberts WG (2008). Antitumor activity and pharmacology of a selective focal adhesion kinase inhibitor, PF-562,271. Cancer Res..

[242] Infante JR (2012). Safety, pharmacokinetic, and pharmacodynamic phase I dose-escalation trial of PF-00562271, an inhibitor of focal adhesion kinase, in advanced solid tumors. J. Clin. Oncol..

[243] Popow J (2019). Highly selective PTK2 proteolysis targeting chimeras to probe focal adhesion kinase scaffolding functions. J. Med. Chem..

[244] Gao, H. et al. Design, synthesis, and evaluation of highly potent FAK-targeting PROTACs. *ACS Med. Chem. Lett.*10.1021/acsmedchemlett.9b00372 (2019). 10.1021/acsmedchemlett.9b00372 PMC7549110 33062164

[245] Rosnet O (1996). Human FLT3/FLK2 receptor tyrosine kinase is expressed at the surface of normal and malignant hematopoietic cells. Leukemia.

[246] Brasel K (1995). Expression of the Flt3 receptor and its ligand on hematopoietic-cells. Leukemia.

[247] Kindler T, Lipka DB, Fischer T (2010). FLT3 as a therapeutic target in AML: still challenging after all these years. Blood.

[248] Pratz KW, Levis M (2017). How I treat FLT3-mutated AML. Blood.

[249] Yamamoto Y (2001). Activating mutation of D835 within the activation loop of FLT3 in human hematologic malignancies. Blood.

[250] Kiyoi H (2002). Mechanism of constitutive activation of FLT3 with internal tandem duplication in the juxtamembrane domain. Oncogene.

[251] Hayakawa F (2000). Tandem-duplicated Flt3 constitutively activates STAT5 and MAP kinase and introduces autonomous cell growth in IL-3-dependent cell lines. Oncogene.

[252] Yokota S (1997). Internal tandem duplication of the FLT3 gene is preferentially seen in acute myeloid leukemia and myelodysplastic syndrome among various hematological malignancies. A study on a large series of patients and cell lines. Leukemia.

[253] Cortes J (2018). Quizartinib, an FLT3 inhibitor, as monotherapy in patients with relapsed or refractory acute myeloid leukaemia: an open-label, multicentre, single-arm, phase 2 trial. Lancet Oncol..

[254] Perl AE (2017). Selective inhibition of FLT3 by gilteritinib in relapsed or refractory acute myeloid leukaemia: a multicentre, first-in-human, open-label, phase 1-2 study. Lancet Oncol..

[255] Odia Y (2016). A Phase II trial of tandutinib (MLN 518) in combination with bevacizumab for patients with recurrent glioblastoma. CNS Oncol..

[256] Fiedler W (2015). A phase I/II study of sunitinib and intensive chemotherapy in patients over 60 years of age with acute myeloid leukaemia and activating FLT3 mutations. Br. J. Haematol..

[257] Shah NP (2013). Ponatinib in patients with refractory acute myeloid leukaemia: findings from a phase 1 study. Br. J. Haematol..

[258] Burslem GM (2018). Enhancing antiproliferative activity and selectivity of a FLT-3 inhibitor by proteolysis targeting chimera conversion. J. Am. Chem. Soc..

[259] Falkenberg KJ, Johnstone RW (2014). Histone deacetylases and their inhibitors in cancer, neurological diseases and immune disorders. Nat. Rev. Drug Discov..

[260] Xu WS, Parmigiani RB, Marks PA (2007). Histone deacetylase inhibitors: molecular mechanisms of action. Oncogene.

[261] Losson H, Schnekenburger M, Dicato M, Diederich M (2016). Natural compound histone deacetylase inhibitors (HDACi): synergy with inflammatory signaling pathway modulators and clinical applications in cancer. Molecules.

[262] Boyault C (2006). HDAC6-p97/VCP controlled polyubiquitin chain turnover. EMBO J..

[263] Dokmanovic M, Clarke C, Marks PA (2007). Histone deacetylase inhibitors: overview and perspectives. Mol. Cancer Res..

[264] Bazzaro M (2008). Ubiquitin proteasome system stress underlies synergistic killing of ovarian cancer cells by bortezomib and a novel HDAC6 inhibitor. Clin. Cancer Res..

[265] Kalin JH, Bergman JA (2013). Development and therapeutic implications of selective histone deacetylase 6 inhibitors. J. Med. Chem..

[266] Kanno K (2012). Overexpression of histone deacetylase 6 contributes to accelerated migration and invasion activity of hepatocellular carcinoma cells. Oncol. Rep..

[267] Bradbury CA (2005). Histone deacetylases in acute myeloid leukaemia show a distinctive pattern of expression that changes selectively in response to deacetylase inhibitors. Leukemia.

[268] Yang K (2018). Development of the first small molecule histone deacetylase 6 (HDAC6) degraders. Bioorg. Med. Chem. Lett..

[269] Wu H (2019). Development of multifunctional histone deacetylase 6 degraders with potent antimyeloma activity. J. Med. Chem..

[270] An Z (2019). Developing potent PROTACs tools for selective degradation of HDAC6 protein. Protein Cell..

[271] Gupta VA (2017). Bone marrow microenvironment derived signals induce Mcl-1 dependence in multiple myeloma. Blood.

[272] Czabotar PE, Guillaume L, Andreas S, Adams JM (2014). Control of apoptosis by the BCL-2 protein family: implications for physiology and therapy. Nat. Rev. Mol. Cell Biol..

[273] Papatzimas JW (2019). From inhibition to degradation: targeting the antiapoptotic protein myeloid cell leukemia 1 (MCL1). J. Med. Chem..

[274] Vogelstein B, Lane D, Levine AJ (2000). Surfing the p53 network. Nature.

[275] Feki A, Irminger-Finger I (2004). Mutational spectrum of p53 mutations in primary breast and ovarian tumors. Crit. Rev. Oncol. Hematol..

[276] Freedman DA, Wu L, Levine AJ (1999). Functions of the MDM2 oncoprotein. Cell Mol. Life Sci..

[277] Wang B (2019). Development of selective small molecule MDM2 degraders based on nutlin. Eur. J. Med. Chem..

[278] Cuadrado A, Nebreda AR (2010). Mechanisms and functions of p38 MAPK signalling. Biochem J..

[279] Cargnello M, Roux PP (2012). Activation and function of the MAPKs and their substrates, the MAPK-activated protein kinases. Microbiol. Mol. Biol. Rev..

[280] Gibson BA, Kraus WL (2012). New insights into the molecular and cellular functions of poly(ADP-ribose) and PARPs. Nat. Rev. Mol. Cell Biol..

[281] Ame JC, Spenlehauer C, de Murcia G (2004). The PARP superfamily. Bioessays.

[282] Cepeda V (2006). Poly(ADP-ribose) polymerase-1 (PARP-1) inhibitors in cancer chemotherapy. Recent Pat. Anticancer Drug Discov..

[283] Zhao Q, Lan T, Su S, Rao Y (2019). Induction of apoptosis in MDA-MB-231 breast cancer cells by a PARP1-targeting PROTAC small molecule. Chem. Commun..

[284] Thorpe LM, Yuzugullu H, Zhao JJ (2015). PI3K in cancer: divergent roles of isoforms, modes of activation and therapeutic targeting. Nat. Rev. Cancer.

[285] Burke JE, Williams RL (2015). Synergy in activating class I PI3Ks. Trends Biochem Sci..

[286] Akinleye A (2013). Phosphatidylinositol 3-kinase (PI3K) inhibitors as cancer therapeutics. J. Hematol. Oncol..

[287] Ciruelos Gil EM (2014). Targeting the PI3K/AKT/mTOR pathway in estrogen receptor-positive breast cancer. Cancer Treat. Rev..

[288] Dey N, De P, Leyland-Jones B (2017). PI3K-AKT-mTOR inhibitors in breast cancers: From tumor cell signaling to clinical trials. Pharm. Ther..

[289] Dubrovska A (2009). The role of PTEN/Akt/PI3K signaling in the maintenance and viability of prostate cancer stem-like cell populations. Proc. Natl Acad. Sci. USA.

[290] Liu P, Cheng H, Roberts TM, Zhao JJ (2009). Targeting the phosphoinositide 3-kinase pathway in cancer. Nat. Rev. Drug Discov..

[291] Zhu J, Hou T, Mao X (2015). Discovery of selective phosphatidylinositol 3-kinase inhibitors to treat hematological malignancies. Drug Discov. Today.

[292] Zhu W (2015). Design, synthesis and docking studies of novel thienopyrimidine derivatives bearing chromone moiety as mTOR/PI3Kalpha inhibitors. Eur. J. Med. Chem..

[293] Shao T (2014). Discovery of 2-methoxy-3-phenylsulfonamino-5-(quinazolin-6-yl or quinolin-6-yl)benzamides as novel PI3K inhibitors and anticancer agents by bioisostere. Eur. J. Med. Chem..

[294] Zheng Z (2016). Discovery and antiplatelet activity of a selective PI3Kbeta inhibitor (MIPS-9922). Eur. J. Med. Chem..

[295] Heffron TP (2011). Rational design of phosphoinositide 3-kinase alpha inhibitors that exhibit selectivity over the phosphoinositide 3-kinase beta isoform. J. Med. Chem..

[296] Li W (2018). Phthalimide conjugations for the degradation of oncogenic PI3K. Eur. J. Med. Chem..

[297] Liu F (2013). Pirin is an iron-dependent redox regulator of NF-kappaB. Proc. Natl Acad. Sci. USA.

[298] Komai K, Niwa Y, Sasazawa Y, Simizu S (2015). Pirin regulates epithelial to mesenchymal transition independently of Bcl3-slug signaling. FEBS Lett..

[299] Dunwell JM, Purvis A, Khuri S (2004). Cupins: the most functionally diverse protein superfamily?. Phytochemistry.

[300] Chessum NEA (2018). Demonstrating in-cell target engagement using a pirin protein degradation probe (CCT367766). J. Med. Chem..

[301] Margueron R, Reinberg D (2011). The Polycomb complex PRC2 and its mark in life. Nature.

[302] Liu YL (2015). Expression and clinicopathological significance of EED, SUZ12 and EZH2 mRNA in colorectal cancer. J. Cancer Res. Clin. Oncol..

[303] Justin N (2016). Structural basis of oncogenic histone H3K27M inhibition of human polycomb repressive complex 2. Nat. Commun..

[304] Kasinath V (2018). Structures of human PRC2 with its cofactors AEBP2 and JARID2. Science.

[305] Gan L (2018). Epigenetic regulation of cancer progression by EZH2: from biological insights to therapeutic potential. Biomark. Res..

[306] Frances Potjewyd, et al. Degradation of polycomb repressive complex 2 with an EED-targeted bivalent chemical degrader. *BioRxiv*, 10.1101/676965 (2019). 10.1016/j.chembiol.2019.11.006 PMC7004250 31831267

[307] Humphries F, Yang S, Wang B, Moynagh PN (2015). RIP kinases: key decision makers in cell death and innate immunity. Cell Death Differ..

[308] Husnjak K, Dikic I (2012). Ubiquitin-binding proteins: decoders of ubiquitin-mediated cellular functions. Annu. Rev. Biochem..

[309] Song Y (2019). Development and preclinical validation of a novel covalent ubiquitin receptor Rpn13 degrader in multiple myeloma. Leukemia.

[310] Kobayashi T, Cohen P (1999). Activation of serum- and glucocorticoid-regulated protein kinase by agonists that activate phosphatidylinositide 3-kinase is mediated by 3-phosphoinositide-dependent protein kinase-1 (PDK1) and PDK2. Biochem. J..

[311] Garcia-Martinez JM, Alessi DR (2008). mTOR complex 2 (mTORC2) controls hydrophobic motif phosphorylation and activation of serum- and glucocorticoid-induced protein kinase 1 (SGK1). Biochem. J..

[312] Vasudevan KM (2009). AKT-independent signaling downstream of oncogenic PIK3CA mutations in human cancer. Cancer Cell..

[313] Malik N (2018). Mechanism of activation of SGK3 by growth factors via the Class 1 and Class 3 PI3Ks. Biochem. J..

[314] Tovell H (2019). Design and characterization of SGK3-PROTAC1, an isoform specific SGK3 kinase PROTAC degrader. ACS Chem. Biol..

[315] Chung AC (2010). Advanced glycation end-products induce tubular CTGF via TGF-beta-independent Smad3 signaling. J. Am. Soc. Nephrol..

[316] Liu Z, Huang XR, Lan HY (2012). Smad3 mediates ANG II-induced hypertensive kidney disease in mice. Am. J. Physiol. Ren. Physiol..

[317] Wang W (2006). Essential role of Smad3 in angiotensin II-induced vascular fibrosis. Circ. Res..

[318] Zhou L (2010). Mechanism of chronic aristolochic acid nephropathy: role of Smad3. Am. J. Physiol. Ren. Physiol..

[319] Wang X (2016). New strategy for renal fibrosis: targeting Smad3 proteins for ubiquitination and degradation. Biochem. Pharmacol..

[320] Heppler LN, Frank DA (2019). Inhibit versus destroy: are PROTAC degraders the solution to targeting STAT3?. Cancer Cell..

[321] Bai L (2019). A potent and selective small-molecule degrader of STAT3 achieves complete tumor regression in vivo. Cancer Cell..

[322] Crew AP (2018). Identification and characterization of Von Hippel-Lindau-recruiting proteolysis targeting chimeras (PROTACs) of TANK-binding kinase 1. J. Med. Chem..

[323] Barbie DA (2009). Systematic RNA interference reveals that oncogenic KRAS-driven cancers require TBK1. Nature.

[324] Muvaffak A (2014). Evaluating TBK1 as a therapeutic target in cancers with activated IRF3. Mol. Cancer Res..

[325] Zhan Y (2015). Development of novel cellular histone-binding and chromatin-displacement assays for bromodomain drug discovery. Epigenetics Chromatin..

[326] Gechijian LN (2018). Functional TRIM24 degrader via conjugation of ineffectual bromodomain and VHL ligands. Nat. Chem. Biol..

[327] de Wispelaere M (2019). Small molecule degraders of the hepatitis C virus protease reduce susceptibility to resistance mutations. Nat. Commun..

[328] Akira S, Takeda K (2004). Toll-like receptor signalling. Nat. Rev. Immunol..

[329] Nunes J (2019). Targeting IRAK4 for degradation with PROTACs. ACS Med. Chem. Lett..

[330] Bassi ZI (2018). Modulating PCAF/GCN5 immune cell function through a PROTAC approach. ACS Chem. Biol..

[331] Swyter S (2018). New chemical tools for probing activity and inhibition of the NAD(+)-dependent lysine deacylase sirtuin 2. Philos. Trans. R. Soc. Lond. B Biol. Sci..

[332] Schiedel M (2018). Chemically induced degradation of Sirtuin 2 (Sirt2) by a proteolysis targeting chimera (PROTAC) based on sirtuin rearranging ligands (SirReals). J. Med. Chem..

[333] Chu TT (2016). Specific knockdown of endogenous tau protein by peptide-directed ubiquitin-proteasome degradation. Cell Chem. Biol..

[334] Lu M (2018). Discovery of a Keap1-dependent peptide PROTAC to knockdown Tau by ubiquitination-proteasome degradation pathway. Eur. J. Med. Chem..

[335] Silva, M. C. et al. Targeted degradation of aberrant tau in frontotemporal dementia patient-derived neuronal cell models. *Elife*. **8**, 10.7554/eLife.45457 (2019). 10.7554/eLife.45457 PMC6450673 30907729

[336] Winter GE (2015). Phthalimide conjugation asastrategy for invivo target protein degradation. Science.

[337] Nabet B (2018). The dTAG system for immediate and target-specific protein degradation. Nat. Chem. Biol..

[338] Reynders, M. et al. PHOTACs Enable optical control of protein degradation. *ChemRxiv*, preprint, https://chemrxiv.org/articles/PHOTACs_Enable_Optical_Control_of_Protein_Degradation/8206688 (2019). 10.1007/978-1-0716-1665-9_17 34432252

